# A comprehensive survey of multi-modality medical image fusion: methods, challenges, and applications

**DOI:** 10.1186/s12880-026-02555-1

**Published:** 2026-07-14

**Authors:** Sa. I. Ibrahim

**Affiliations:** Faculty of Computers and Informatics, Suez Canal University, Information Systems Department, Ismailia, Egypt

**Keywords:** Medical image fusion, Multi-Scale Transform, Evaluation metrics, Computed Tomography, Magnetic Resonance Image

## Abstract

Medical image fusion (MIF) is the process of fusing two medical pictures from different modalities into one image. This technology tries to produce a fused output image from two source images that contain more effective and relevant information. This image is used in the healthcare industry, specifically for disease diagnosis. The main challenge is using a single image modality to diagnose diseases accurately. The fused image includes spectral and structural information for the source images to help doctors with disease diagnosis problems. Positron emission tomography (PET), magnetic resonance imaging (MRI), computed tomography (CT), and single photon emission computed tomography (SPECT) are some of the medical imaging modalities. Each modality has its benefits and drawbacks. Researchers have presented different MIF techniques that obtain high fusion results in the MIF field. This paper is a comprehensive survey of multiple state-of-the-art MIF techniques in the spatial and transform domains. It also discusses the main MIF evaluation metrics. Finally, quantitative and qualitative evaluations for some of these techniques are obtained.

## Introduction

Nowadays, massive advancements in the healthcare sector have resulted in the development of several imaging sensors that have enhanced medical decision-making. Diagnosis situations often require viewing several structures of an individual’s body, which are typically not visible with a single modality. Therefore, the data from multiple sensors may be merged to create a new image that may further inform specialists by providing complementary information [1]. Image fusion is a promising research area in object detection, medical diagnosis, biomedical research, multimedia analysis, computer vision, and remote sensing [2]. Image fusion combines valuable information from one or more registered images to generate a fused image with complementary information without distortions, decreases noise, and eliminates image redundancy [3, 4]. The combination of several modalities delivers more information than a single modality, producing more accurate results. The resulting image should also retain the relevant information and decrease ambiguity from the original images [5]. As previously stated, image fusion has numerous goals, such as creating an effective fusion model that merges multiple image modalities into a single output image to help radiologists in diagnosing diseases and decision-making. It also improves spatial resolution and enhances non-visible elements in either single mode.

Medical imaging has played a critical role in healthcare domains such as biomedical research, treatment, and medical diagnosis [2, 6]. In medical imaging, there are numerous modalities such as CT, SPECT, MRI, PET, Ultrasound(US), Optical Coherence Tomography (OCT), and Contrast-Enhanced Ultrasound (CEUS). These modalities were collected from various sensors and addressed particular organ properties. It is hard to gather all the data from a single imaging modality to ensure the clinical accuracy of the precise medical diagnosis [2, 6].

CT generates cross-sectional images using X-rays and tissue attenuation coefficients. CT is the preferred method of identifying acute brain blood, skull injuries, brain edema, and hydrocephalus [7]. CT gives great visibility of bone structures and acute blood cells, but it has poor soft-tissue contrast [7]. CT has great spatial resolution (0.5–5 mm) and a quick acquisition time [8]. In fusion situations, CT is frequently used with PET or MRI to provide anatomical context for functional or soft-tissue data [7].

MRI uses hydrogen protons to produce images with high soft-tissue contrast, making it the standard for brain imaging [7]. MRI offers superior soft-tissue contrast [7] with long acquisition times (30–60 minutes), and high cost. In fusion, MRI is combined with PET or CT [9]. Different MRI sequences highlight specific tissue features [9]. T1-weighted (T1-w) images show anatomical details, help distinguish gray and white matter, and reveal contrast-enhanced lesions [9]. T2-weighted (T2-w) images are highly sensitive to water and edema, so they are used to detect brain tumors [9]. Fluid-Attenuated Inversion Recovery (FLAIR) reduces the signal from cerebrospinal fluid, which identifies periventricular diseases such as multiple sclerosis plaques [9]. Diffusion-weighted imaging (DWI) measures water movement and is important for detecting early acute ischemic stroke, as it can show changes within minutes of symptom onset [9].

PET uses gamma rays generated by positron-emitting radiotracers to represent metabolic and molecular activities [7]. PET is used in brain imaging to detect epileptic foci, characterize brain cancers, and diagnose neurodegenerative illnesses, including Alzheimer’s and Parkinson’s [7]. However, PET has low spatial resolution (usually 4–6 mm) and delivers very limited anatomical information [8]. PET is usually used with CT or MRI to precisely identify metabolic disorders. PET-MRI is the ideal method for brain imaging because it combines functional PET data with MRI, which provides better soft tissue contrast [9].

SPECT, like PET, employs gamma-emitting radiotracers to assess brain blood flow and molecular behavior [7]. SPECT is commonly used in brain imaging to monitor brain blood flow in stroke, diagnose diseases that distinguish Alzheimer’s disease from frontotemporal disease, and identify epileptic foci [7]. SPECT has a relatively limited spatial resolution (7–15 mm) and is commonly combined with CT or MRI for anatomical identification [8]. It costs less than PET [10].

Ultrasound (US) employs high-frequency sound waves to create real-time images. Its advantages include accessibility, low cost, absence of ionizing radiation, and real-time capabilities [11]. Ultrasound is effective for guiding tissue samples, vascular investigations, and obstetric imaging [7]. Poor transmission through the skull limits the use of ultrasound for brain imaging [11]. US can be used with CT or MRI for intervention guiding [7].

Optical Coherence Tomography (OCT) utilizes slow-coherence interferometric techniques to capture ultra-resolution cross-sectional images of tissue substructure [12]. OCT has micrometer resolution (1–15 $${\mu}$$m) but a low penetration depth ($$ <1$$ cm) [12]. It is most used in retinal and intravascular imaging [13]. OCT is being utilized in combination with other modalities to diagnose ocular and endovascular disorders [7].

Contrast-Enhanced Ultrasound (CEUS) uses microbubble contrast agents to improve us images and allow real-time evaluation of blood flow [7]. It is mainly used to identify liver lesions, view blood vessels, and assess tumor blood flow [7]. CEUS can detect vascular lesions with super sensitivity and does not use ionizing radiation [7].

A comparison of common medical imaging modalities is in Table 1. There are some defects in each type of these images, so it is very important to perform an image fusion technique to solve these defects.

**Table 1:** Comparison of major medical imaging modalities

Modality	Image Formation	Strengths	Limitations	Fusion combinations
(a) CT	X-ray attenuation [14]	Great spatial resolution (0.5–5 mm) [8]; quick acquisition time; excellent for bone and acute blood cells [7]	Ionizing radiation [9]; poor soft-tissue contrast [7]; more artifacts	PET-CT [7]; MRI-CT [9]
(b) MRI	Hydrogen protons [14]	High soft-tissue contrast [7]; without ionizing radiation [9]; Multiple sequence (T1, T2, FLAIR, and DWI) [9]	High acquisition time (30–60 min) [9]; high cost [7]; insufficient for real-time [11]	PET-MRI [9]; MRI-CT [7]
(c) PET	Gamma detection [14]	Metabolic/Functional imaging [7]; high sensitivity [8]; all body imaging	High radiation; Low spatial resolution [8]; limited anatomical information [7]; high cost [9]	PET-CT [7]; PET-MRI [9]
(d) SPECT	Gamma detection [14]	Brain perfusion [7]; heart muscle perfusion; lower cost than PET [7]	Ionizing radiation; Very limited spatial resolution (7–15 mm) [8]; limited anatomical detail [7]	SPECT-CT [7]; SPECT-MRI [13]
Ultrasound (US)	High-frequency sound wave (1–20 MHz) [11]	No ionizing radiation; real-time imaging; portable; low cost [7]	Poor transmission through bone; finite depth (12–15 cm) [11]	US-CT [7]; US-MRI [9]
OCT	Slow-coherence interferometric techniques [12]	No ionizing radiation; real-time; micrometer resolution (1–15 $\mu$m) [12]	Low penetration depth ($<$1 cm) [12]	OCT-US [7]
CEUS	Microbubble contrast agents to improve us [11]	Real-time perfusion of blood flow; no ionizing radiation [7]; great sensitivity for detecting vascular lesions;	Requires contrast medical aid; low quantification	CEUS-CT; CEUS-MRI [7]

**Fig. 1:**
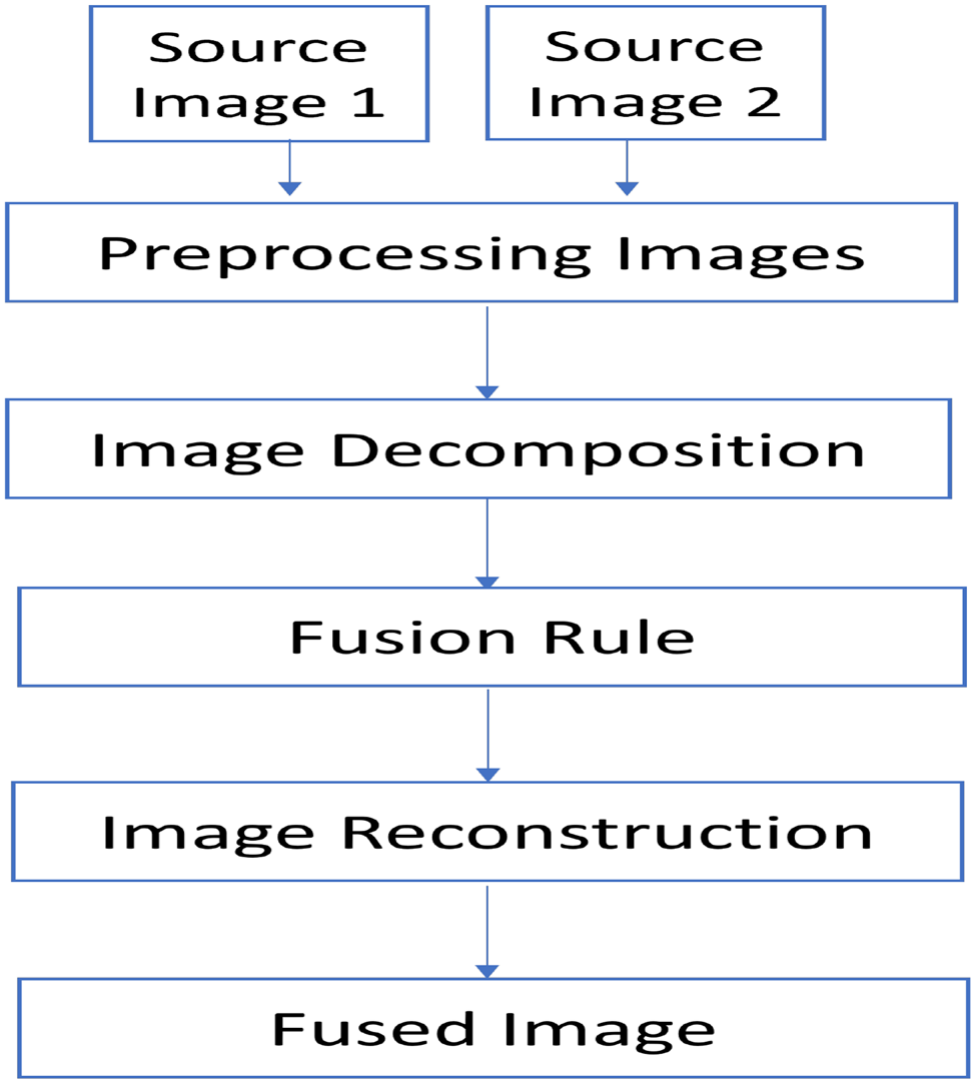
Basic image fusion process [21]

Medical image fusion (MIF) integrates multiple images from various modalities to generate the fused image. MIF contains more vital information that is applicable for clinical research. It also helps doctors make decisions in the disease diagnosis area. The key benefit of MIF is that it reduces the difficulty of identifying various diseases. Most MIF methods in the transform domain use multiscale transform (MST). Non-subsampled contourlet transform (NSCT) [15, 16], convolutional neural networks (CNN) [17], Laplacian pyramid (LP) [18], and non-subsampled shearlet transform (NSST) [19] are MST-based image fusion methods. Image decomposition, fusion rules, and image reconstruction are the three fundamental processes in every fusion method [4, 20]. Figure 1 illustrates the image fusion process.

- *Preprocessing:* The process of improving the source images, including removing noise from the source images and enhancing the contrast of the source images. This steps aim to unity image size, data format and resolution.
- *Image Decomposition:* decomposing two source images using decomposition methods like the NSCT, NSST, and others then producing sub-images coefficients named low and high-frequency coefficients.
- *Fusion Rule:* is the process of merging low and high-frequency coefficients using any fusion rule such as Principal Component Analysis (PCA), Pulse Coupled Neural Networks (PCNNs), etc. The results of this step are low fused and high fused.

**Fig. 2:**
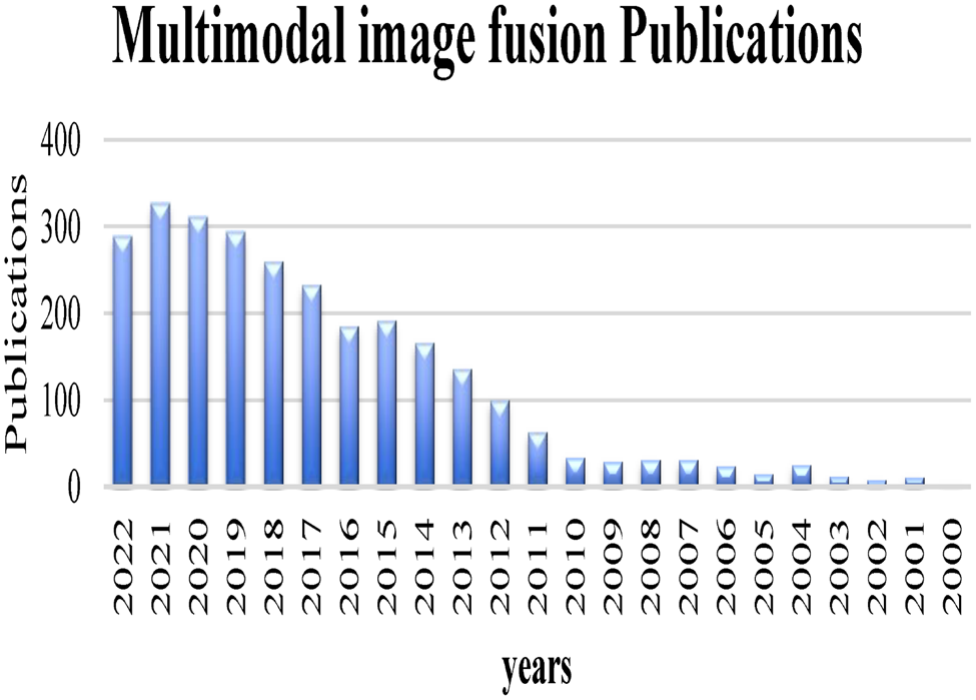
MIF publications [22]

Image fusion has some advantages and disadvantages, all of these can be mentioned in the following Table 2. Research on MIF has rapidly increased to illustrate the importance of multimodal MIF. Figure 2 illustrates the number of published research papers on multimodal MIF from 2000 through the third quarter of 2022, counted by PubMed [22, 23].

**Table 2:** Advantages and disadvantages of image fusion

Advantages	Disadvantage
– Enhance the information of the fused image by incorporating the most valuable features from the source images.	– The computational of the fusion process may be complex and require more complex algorithms with significant computational resources.
– The fused images have lower noise, higher resolution, and higher contrast than the single input image.	– The fused image may be low quality based on the quality of the input images from various sensors.
– The fused image with high performance that aids doctor to make more accurate decision in various areas such as medical imaging and remote sensing.	– The fused image resullted from poor fusion techniques may suffer from artifacts and may loss some useful information.
– Remove the reduent information.	– The fusion process in some fusion techniques may consume more time.

### Motivations

MIF techniques have developed as an important option for overcoming the limits of separate imaging modalities, resulting in improved diagnosis, treatment planning, and clinical decision-making. Every modality, such as CT, MRI, PET, or SPECT, delivers distinct information; for example, CT specializes in structural detail, MRI shows soft tissue contrast, and PET detects metabolic activity. However, dependence on a single modality frequently yields partial or insufficient diagnostic data.

The primary motivations for this study are to summarize conventional and new research papers in the area of MIF. It also assists researchers in selecting the most effective way for merging medical images that has the following characteristics: high efficiency, high spatial resolution preservation, and minimal color distortion. Using the best fusion method can help doctors diagnose diseases more precisely. Furthermore, combining medical images from many multimodal sources into a single fused output image. This enhanced image quality allows healthcare professionals to observe and interpret medical details more clearly and precisely.

### Contribution

This manuscript presents an original survey of multimodality medical image fusion techniques, focusing on methods, challenges, and applications. This survey provides a comprehensive review of the latest advancements in MIF techniques, focusing on methods, applications, and challenges. It categorizes existing approaches into traditional methods, such as pixel- and feature-based techniques, and modern approaches in machine learning. The contribution lies in critically analyzing these methods to identify strengths and limitations. Additionally, the survey highlights the role of quality metrics in evaluating fusion performance. This review serves as a valuable resource for researchers and practitioners seeking to enhance the effectiveness of medical image fusion in clinical practice.

The rest of the paper is organized as follows. Section 2 includes the image fusion levels and modalities. Section 3 discusses medical image fusion techniques and related work. Section 4 discuss the most common used datasets in the MIF area. Section 5 presents the evaluation metrics to assess the performance of fusion techniques. quality measures. The MIF challenges are summarized in Sect. 6. The applications of medical image fusion in machine learning domain are discussed in Sect. 7. Finally, We conclude and summarize whole the paper in Sect. 8.

## Image fusion levels and modalities

### Low-frequency and high-frequency subbands

- **Low-frequency subbands (also known as LFCs)** represent slowly varying components such as global contrast, large-scale anatomical features, smooth variations, and background light, accounting for 80-95% of the image’s signal energy [21, 24]. The low-frequency region of the image displays the body information, such as the object’s contour and the fundamental component area [25].
- **High-frequency subbands (also known as HFCs)** represent rapidly varying components such as edges, sharp transitions, fine textures, noise, and tiny anatomical features. They account for just 5-20% of the signal energy yet are perceptually significant for human vision and important for diagnostic detail [21, 24]. The high-frequency represents the image’s detailed information, such as texture and object edges [25]. while focusing on low-frequency information produces a blurred image with few fine details, whereas high augmentation of high-frequency components might remove noise or add artifacts [10, 24].

### Image fusion levels

There are three levels of image fusion: pixel-level fusion, decision-level fusion, and feature-level fusion [26].

**Fig. 3:**
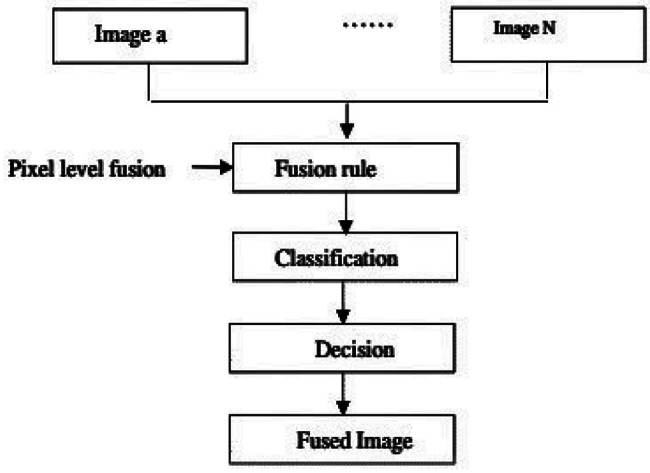
Pixel-level fusion diagram [27]
  *Spatial domain methods:* recognize image pixels in the source images, spatial domain methods generate the fused image. The information from the original images is retained in the fused images using these methods. The core advantage of these methods is their short computing time. On the other hand, they provide low-contrast pictures with color and spatial distortion [3, 5]. Principal component analysis (PCA) and weighted averages are examples of spatial domain methods.
  *Transform domain methods:* transforming the source images into the frequency domain, transform domain methods reconstruct the fused image. The low and high-frequency coefficients are inputs to the fusion rule. Inversing the transformation yields the fused image. The transform domain’s key advantages are that it avoids distortion and is more efficient than spatial domain methods [5].
- *Feature-level fusion techniques:* retrieve the source images’ features like shapes, textures, lengths, edges, and directions to generate a significant descriptive image. These features are retrieved individually from every input image then the fusion method is performed depending on these features from the input image [28]. This fusion type is helpful for disease diagnosis but is more complex than pixel-level fusion. Region-based fusion is an example of feature-level fusion. Figure 4 shows the Feature-Level fusion.

- *Pixel-level fusion techniques:* combine the pixel information from the source image to generate an informative fused image. It is a simple and easy fusion type but is still limited to doctors’ ability to diagnose diseases. Figure 3 describes the pixel-level fusion.

**Fig. 4:**
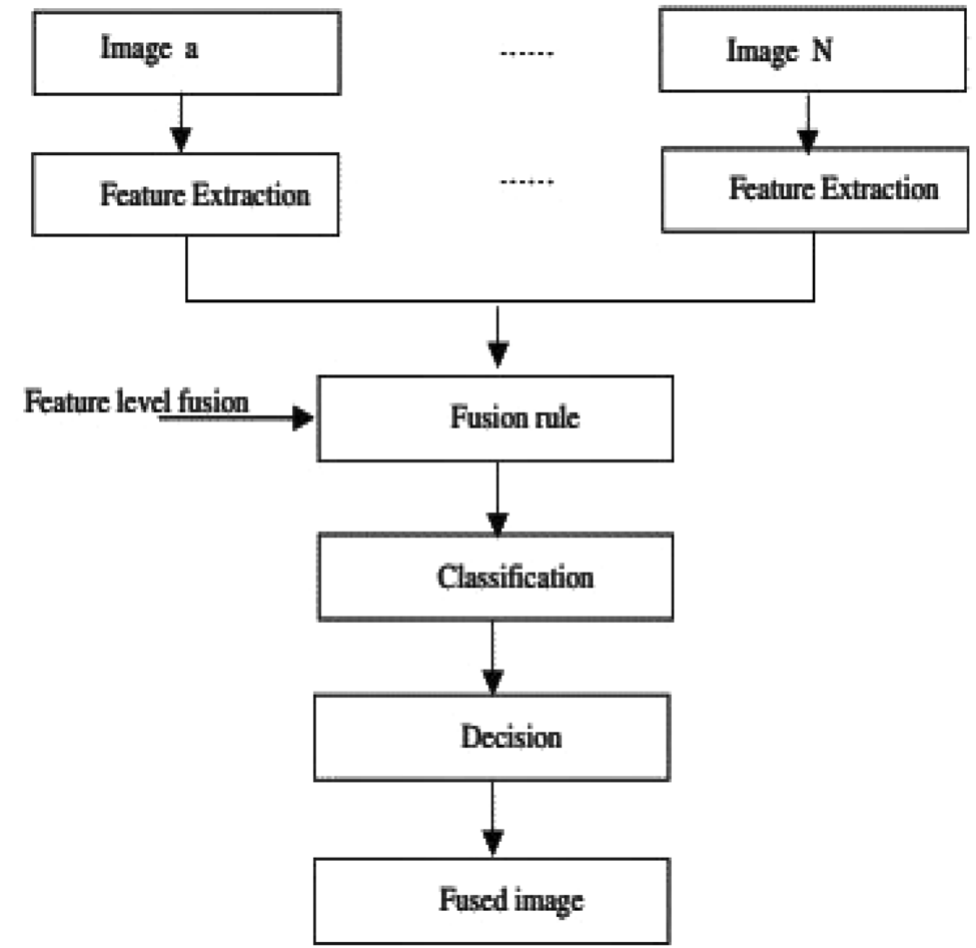
Feature-level fusion diagram [27]
- *Decision-level fusion techniques:* merge the higher-level groupings of the outcomes of many methods to obtain the decision for the fusion process. Each image is treated independently before being sent into the image fusion method. Dictionary learning is a popular method for decision level MIF [28]. This deals with different modalities and data types. The main defect of these type of fusion techniques is may be complex. Figure 5 shows the decision-level fusion.

**Fig. 5:**
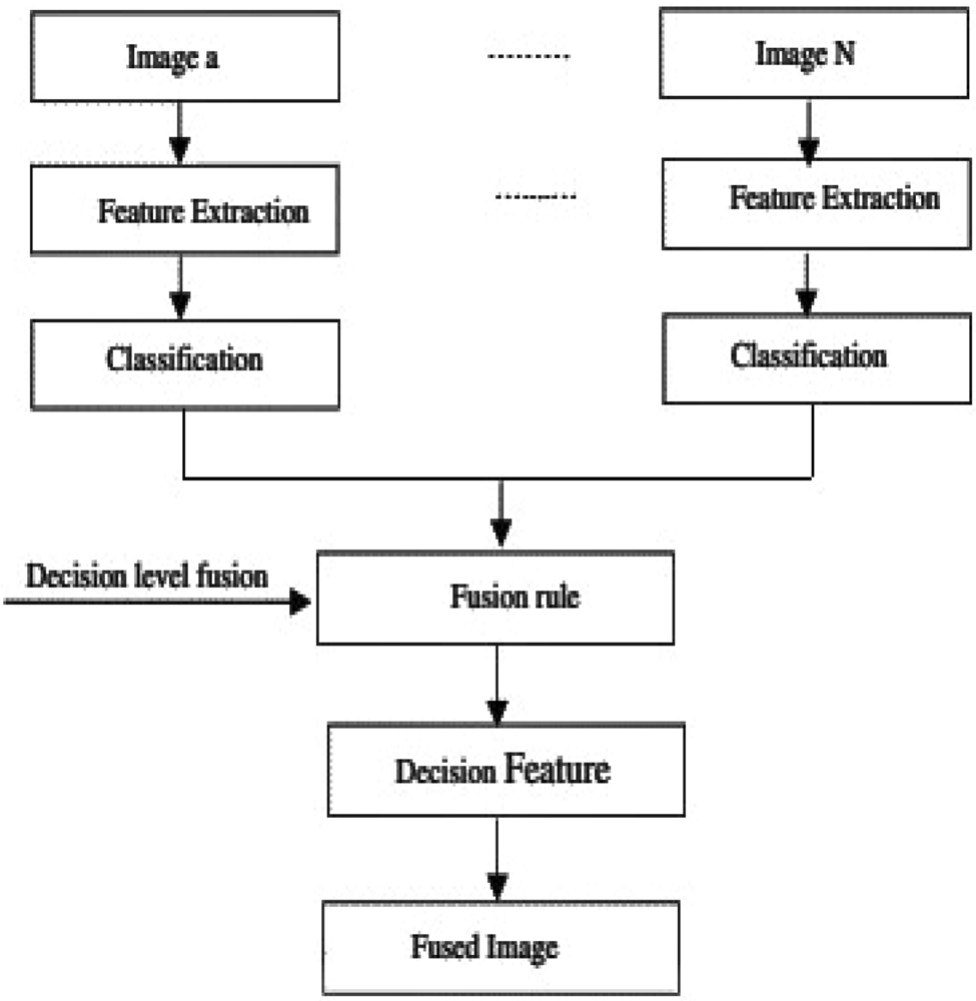
Decision-level fusion diagram [27]

### Image fusion modalities

Image fusion is used in different areas depending on the type of images acquired, like multimodal image fusion, multi-focus image fusion, multi-sensor image fusion, multi-view image fusion, and visible and infrared image fusion. All of these are listed below:

- *Multimodal Image Fusion:* combines structural information from MRI and CT modalities in addition to functional information from PET and SPECT modalities to generate a single image that incorporates complementary and additional information from the source images.
- *Multi-focus Image Fusion:* this method combines two images with opposing left- and right-focused scenes.
- *Multi-sensor Image Fusion:* images from multiple sensors are combined.
- *Multi-view Image Fusion:* this integrates images from separate camera sensors at the same time.
- *Visible and Infrared Image Fusion:* is one in which one image contains visible data and the other has infrared data. Both images are from the same scene [3].

## Medical image fusion techniques

The main type of image fusion is multimodal medical image fusion (MMIF), which combines or fuses multiple images from one or more imaging modalities to increase image quality while keeping essential information. The precise information collected from the combined images allows for effective identification of the position of the disease and diagnosis of diseases. The essential MMIF mainly focus on four image modalities: MRI-CT, MR-T1/MR-T2, MRI-PET, and CT-SPECT.

**Fig. 6:**
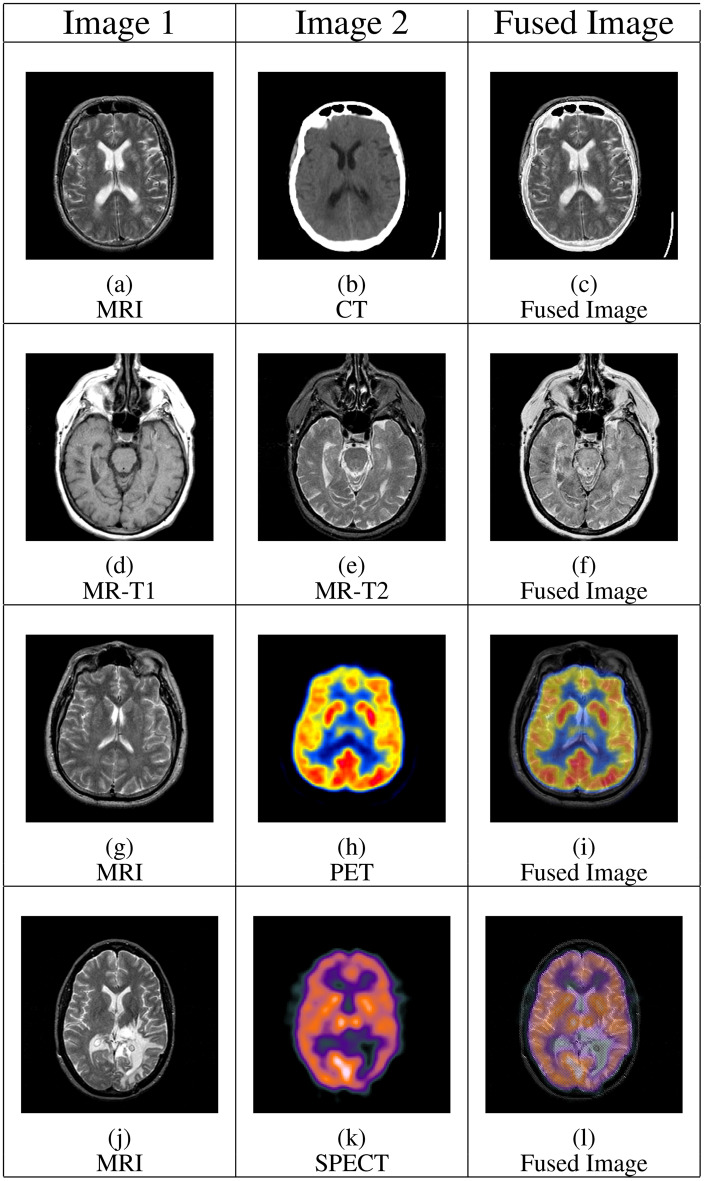
Examples of multimodal medical image fusion (MMIF)

Figure 6 shows the fusion examples and fusion outcomes. This figure provides four instances of medical image fusion (MIF). The first example combines the MRI from Fig. 6a, which describes the soft tissue information, with the CT scan, which contains the hard tissue information from Fig. 6b, creating the fused image in Fig. 6c. The second example combines the MR-T1 in Fig. 6d with the MR-T2 in Fig. 6e, generating the fused image in Fig. 6f. The third example combines the MRI scan to deliver the structured soft information in Fig. 6g with the PET scan to detect the functional information in Fig. 6h, generating the fused image in Fig. 6i. The final example merges the MRI image in Fig. 6j and the SPECT image in Fig. 6k then the result image in Fig. 6l illustrates anatomical activity and blood flow. MIF integrates multiple images from various modalities to generate the fused image. MIF contains more vital information that is applicable for clinical research. It also helps doctors make decisions in the disease diagnosis area. MIF methods have two types spatial domain and frequency domain methods. This section discusses these methods in the spatial and frequency domains. We also list the benefits and limitations of these methods.

### Spatial domain methods

MIF used spatial domain methods in early research. These fusion methods are simple. In the spatial domain, pixels of source images are processed directly instead of the transform coefficients. Pixels are selected based on some measure, then combined using nonlinear or linear operations. The fused image in this domain contains a spatial distortion.

**Fig. 7:**
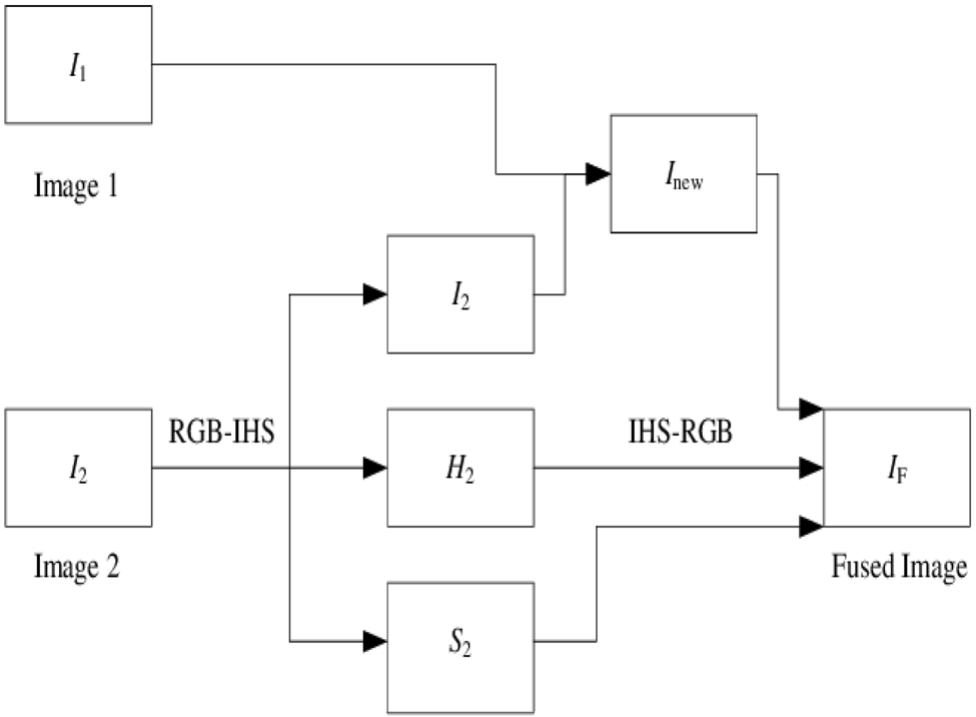
IHS based image fusion process [38]

This section addresses methods for merging medical images in the spatial domain. Those fusion methods involve the principal component analysis method (PCA), the Intensity Hue Saturation (IHS), the minimum, the average, and the maximum fusion. All of the above are listed below:

- *Minimum Fusion* This method is similar to the maximum fusion method, except that the minimum pixel intensity value is determined to produce the fused image [28].
- *Average Fusion* In this method, the corresponding pixels’ averages are computed from the input images to create the fused image. It is simple and clear to execute [29].
- *Maximum Fusion* This method obtains the highest pixel intensity values for the input images and uses them for creating the fused image [28].
- *PCA Fusion* is one of the popular fusion techniques. It seeks to convert the correlated variables into non-correlated variables. It reduces the dimensional information to a lower dimension [30]. PCA can cause some deterioration in spatial domain, So it is not efficient for medical image fusion.
  He et al. [31] proposed a novel image fusion method based on the PCA and IHS transform. The proposed method merges the PCA and IHS features to retain functional and spatial information. Simulation results indicate that the proposed fusion method is high performance than the PCA and discrete wavelet transform (DWT) methods.
  Krishn et al. [32] proposed PCA and ridgelet transform for fusing medical images. The ridgelet transform decomposes the source images. PCA employs a fusion rule to enhance the spatial resolution. Results indicate that the proposed method has high fusion metrics values’. It is also more accurate for medical diagnosis than other fusion methods.
  Himanshi et al. [33] presented an improved method for fusing MRI and CT images depending on PCA and the curvelet transform. The curvelet transform decomposes the source images. The presented method merges the curvelet transform feature for detecting edges with the dimensionality reduction of PCA. Experimental results denote that the presented method accurately fused MRI and CT images while preserving relevant information from the source images using the structural similarity index (SSIM) and the fusion factor (FF) metrics.
  J et al. [34] presented cascaded PCA in shift-invariant wavelet transforms to fuse medical images. The wavelet transform employs shift-invariant features to obtain the image’s phase information. PCA selects valuable information using eigenvalue decomposition. Results show that the presented method improves exact edge details. It also minimizes distortions, unwanted features, and artifacts. Bashir et al. [35] proposed an MMIF model using both PCA and Stationary Wavelet Transform (SWT). This model was evaluated using various medical images. Results show that PCA achieved better performance with multi brightness levels and contrast. SWT had better performance when the images were multi-sensor and multimodal images. The fusion matrices are tested on various sets of images to evaluate the proposed model performance.
  Ghandour et al. [36] proposed an MMIF method using the principal component analysis network (PCANET). This method aims to extract features from medical images. The PCA filter splits the source images into image features and the nuclear norm to construct the activity level maps. The proposed method can preserve the source image details.
- *IHS Fusion* It is commonly used for fusing remote sensing images. This method divides the RGB multispectral image into intensity, hue, and saturation elements. A fused image is created by converting the IHS elements to RGB [37]. The process of image fusion using IHS is described in the Fig. 7.

### Transform domain methods

The transform domain converts the input images from the time or spatial domain to the frequency domain. Transform domains are mainly used in MIF and vary depending on the MST methods. These solve the spatial domain problem while capturing salient features. There are several methods for fusing medical images in the transform domain: Laplacian Pyramid (LP), Discrete Wavelet Transform (DWT), Non-Subsampled Shearlet Transform (NSST), and Non-Subsampled Contourlet Transform (NSCT). All of these methods are discussed below.

#### LP fusion method

The LP method is one of the pyramid decompositions that commonly fuse medical images. This method generates spectral information. On the other hand, it faced a blocking effect, causing the loss of relevant data and further artifices [1, 3]. The LP-based image fusion includes three stages: LP decomposition, fusion rule, and LP reconstruction. LP first decomposes source images. Second, merge coefficients using any fusion rule, such as minimum, maximum, or average fusion. Third, the inverse of the LP method reconstructs the final image.

Sahu et al. [39] implemented the MIF method based on the LP and the Discrete Cosine Transform (DCT). The decomposition step for the CT and MR images using the LP method. The DCT method compresses the source image to minimize the computational time of the decomposition. It also overcomes the Lp artifacts problem. Use the average fusion rule and then apply the inverse of the LP method to construct the final image. The fused image produced by the implemented method has good quality and contains a lot of edge information.

Li et al. [40] presented a new fusion method using sparse representation (SR) and sum-modified Laplacian (SML) in the LP domain. The LP method transforms the source images into low-pass and high-pass subbands. The SR and SML merge the low- and high-pass subbands, respectively. This paper applies four sets of medical images. Results show that the fused images have superior brightness and preserve image information compared to alternative fusion methods.

Liu et al. [41] implemented the MIF method depending on the Convolutional Sparse Representation (CSR) and the LP method. In this paper, the LP method decomposes pre-registered MRI and CT images to achieve base layer and detail layers. Then, the CSR method merges the base layer, whereas the max-absolute method combines the detail layers. The inverse of the LP method produces the fused image. The applied method benefits by extracting the texture information from the input images without decreasing the fused image contrast. The comparative tests reveal that the implemented method outperforms the CSR method in objective and subjective measures.

Li et al. [42] presented the MMIF method based on the Laplacian re-decomposition (LRD) method. This method achieves low-subband images with redundant and complementary information for the high-subband images. The overlapping domain-based fusion (OD) issue obtains redundant information, pixel energy, and image details. On the other hand, non-overlapping domain-based fusion (NOD) retains the domain information and fuses the complementary information. The inverse of the LRD method generates the final image. Evaluation results reveal that the presented method achieves high performance in both quantitative and qualitative terms.

Wang et al. [16] presented the adaptive sparse representation (ASR) and the LP method to fuse medical images. The medical images are decomposed into four images using the LP method. Then use the ASR method to merge the four resulting images. The final image is created using the inverse of the LP method. The comparison reveals that the presented method combines medical images while retaining detailed, structural, and edge information from the input images without producing any distortions. It also enhances the contrast of the final image compared to the traditional methods.

Li et al. [43] proposed an LP-SR method with guided filtering for fusing infrared and visible images. The advantages of this method are low computational time, no training required, and the ability to preserve edges while reducing noise. However, a limitation is that it is not sufficient for medical images, restricting its application to infrared-visible and multi-focus images. To evaluate the method, the TNO, Lytro, and MFI-WHU datasets were used for both color and grayscale images.

#### DWT fusion method

**Fig. 8:**
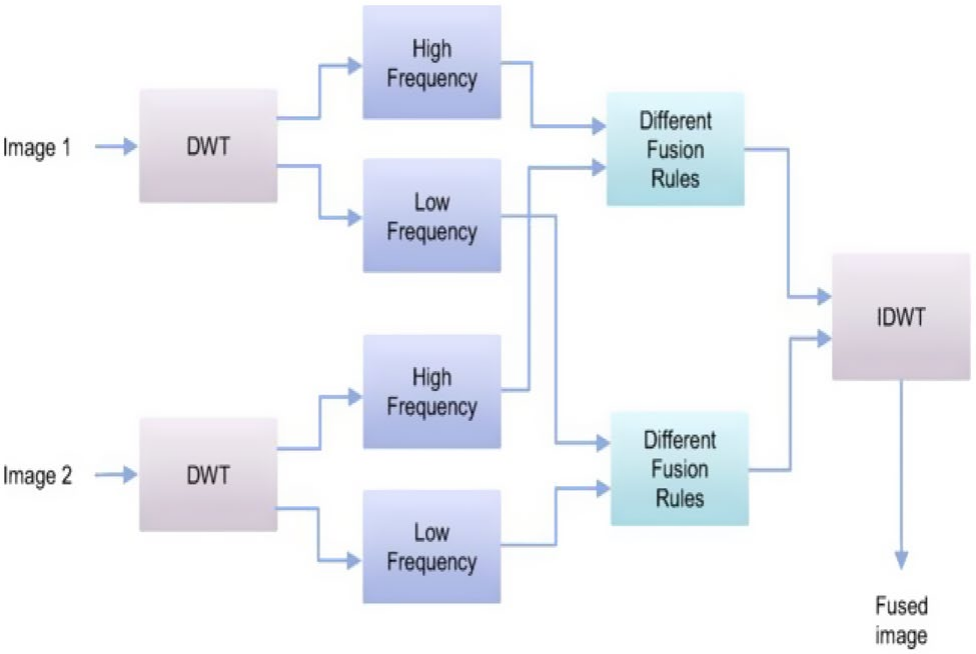
DWT based image fusion [28]

The DWT is a transform domain-based image fusion method. This method addresses the difficulties of the PCA method in the visual and quantitative fusion effect [5]. The decomposition in this method consists of low-and high-frequency subbands. Both subbands are combined using any fusion method. Then the inverse of the DWT method creates the final image. It reduces spatial distortions from the merged image. The main steps of DWT-based image fusion are shown in Fig. 8. DWT-based fusion produces fused images with some artifacts.

Teng et al. [44] proposed a wavelet transform for fusing medical images. The absolute value maximization method fuses the low-frequency subbands, while the weighted averaging method fuses the high-frequency subbands. The proposed method uses CT/MRI images and PET/MRI images. Results reveal that the proposed method improved the properties of the images while retaining image data.

Bhavana et al. [45] applied DWT to fuse both the MRI and PET images. The applied method starts with preprocessing input images to remove noise from these images and uses a Gaussian filter to smooth the input images. The DWT decomposes the PET intensity elements and the MRI to generate high and low-frequency coefficients. The averaging method combines high and low-frequency coefficients. The inverse of DWT produces the fused image. Results show that the applied method reduces color distortion and retains all anatomical information.

Soundrapandiyan et al. [46] presented an accurate MIF based on intuitionistic fuzzy sets (IFS) and DWT methods. In this paper, DWT decomposes the CT and MR images to obtain low and high subbands. The combining of low and high subbands is achieved in two steps. In the first step, average fusion combines low subbands, whereas maximal fusion combines high subbands. In the second step, entropy fusion combines the high subbands, whereas maximum fusion combines the low subbands. The inverse DWT method generates the fused images. The merged pictures are then transformed into the fuzzy domain using the IFS technique, yielding the final image. The results show that the presented method achieved higher contrast in tumor detection than other methods on objective and subjective measures.

#### NSST fusion method

**Fig. 9:**
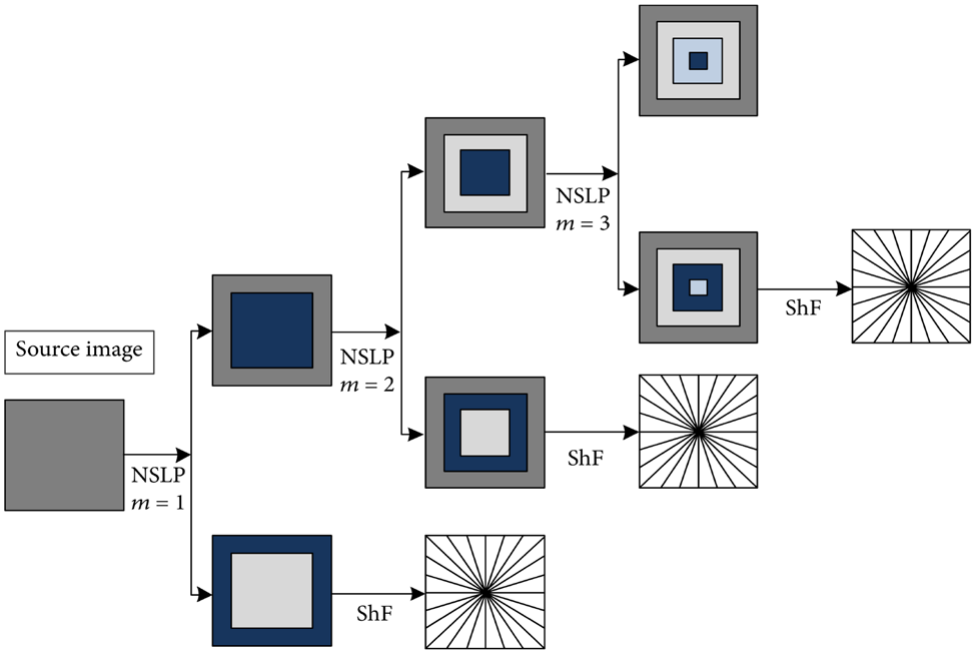
NSST decomposition level [5]

In the field of image processing, the NSST is employed. This MIF method solves the contourlet and the shearlet issues like pseudo-Gibbs phenomena and shift-invariant [47]. The NSST is better than the NSCT in complexity and sparse representation aspects. The shearlet transform filter (ShF) and the non-subsampled Laplacian pyramid (NSLP) filter are being used by NSST to decompose the original images. The NSLP is responsable for decomposing the orignal image into high- and low-frequency components. Figure 9 describes the NSST three-level decomposition. The NSST fusion method is lower computational time and higher sensitivity than the NSCT fusion method.

Ganasala et al. [48] proposed the NSST method for fusing medical images. The high- and low-frequency subbands are given from the source images by using the NSST method. The sum of variations in squares combines the low-frequency subbands to retain detailed information about the input images. The activity level parameters fuse high-frequency subbands while preserving edge information. The combined subbands use the inverse NSST method to generate the final image. The evaluation of the proposed method uses nine groups of medical images for several diseases. The proposed method has higher performance for merging CT-MRI and SPECT-MRI when compared with the NSCT and NSST methods.

Jin et al. [47] applied a MIF novel using a simplified pulse-coupled neural network (S-PCNN) and the NSST method. The NSST decomposes the CT and MR images into high and low-frequency coefficients. The color image is converted into hue-saturation-value (HSV) color space, and then the NSST method decomposes the V component of this image. S-PCNN and intersecting cortical models (ICM) join the high and low-frequency coefficients. The inverse of both NSST and HSV creates the fused image. The image resulted from the applied method involves functional and structural image information.

Yin et al. [49] used the parameter-adaptive pulse-coupled neural network (PAPCNN) method and the NSST method to merge the medical images. First, the source images were decomposed by the NSST method to produce high-frequency and low-frequency bands. Second, the weighted sum of eight neighborhood-based modified Laplacian (WSEML) combines the low-frequency bands. On the other hand, the high-frequency bands were merged by the PAPCNN method. Finally, reconstruct the output image using the inverse NSST method. Evaluation results show that the used method achieves high performance in the MIF area. The benfit of the used method is very fast with low iterations.

Gai et al. [50] demonstrated an MMIF method using the Improved Sparse Representation (ISR) and the Edge Preservation-Pulse Coupled Neural Network (EP-PCNN) in the NSST domain. The medical images are decomposed by the NSST method. The ISR method combines the low-frequency subbands. On the other hand, the EP-PCNN method combines the high-frequency subbands. The inverse NSST method is applied to recreate the fused image. Assessment results show that the given fusion method outperforms in both objective and subjective evaluations.

Vanitha et al. [51] suggested the MIF method based on the spatial frequency (SF) and the PAPCNN method in the NSST domain. The medical images are decomposed into frequency coefficients using the NSST. The high-frequency coefficients are fused using SF and PAPCNN. The maximal rule combines the low-frequency coefficients. The creation of the final image uses the reverse NSST method. The suggested fusion method’s performance is validated using fusion metrics such as Entropy (EN), Mutual Information (MI), SD, and so on. The results indicate that the suggested method combined medical images with retained edge and detail information.

Mei et al. [52] proposed the MMIF method using NSCT and adaptive PCNN for combining medical images. This MMIF method uses the NSST to decompose the input images into low- and high-frequency subbands. Then use the combination of the Sum of Directional Gradients (SDG) and the PCNN model to fuse the low subband frequency coefficients. On the other hand, the high subband frequency coefficients are obtained by merging the local energy and the global gradient. The final fused image is generated using the inverse of the NSST transform. The proposed method is compared using the combinations of the NSCT transform. The main advantage of this method is that it achieves better results with low computational time. The defect of this method uses only CT and MRI images.

Vanitha et al. [53] proposed an MMIF method using the PA-PCNN and energy attribute in the NSST domain. The NSST transformed the input images into low- and high-subband coefficients. The energy attribute fuses the low subband coefficients, and the PA-PCNN fuses the high subband coefficients. The fused image is obtained by applying the inverse NSST to the fused coefficients. The proposed technique is carried out on CT, MRI, PET, and SPECT images. The proposed method uses the fused image in the disease treatment. On the other hand, some edges cannot be detected accurately in the MRI-PET and MRI-SPECT modalities.

Guo et al. [54] presented an MMIF method using the MI and CSR methods. The original images were converted into a single high- and low-frequency subband using the NSST method. MI and CSR methods fuse low-frequency and high-frequency subbands, respectively. In this paper, analysis of two groups of medical imaging modalities are used: CT/MRI and MRI/SPECT. Results demonstrate that the performance of the presented fusion method is better than the other six fusion methods.

#### NSCT fusion method

**Fig. 10:**
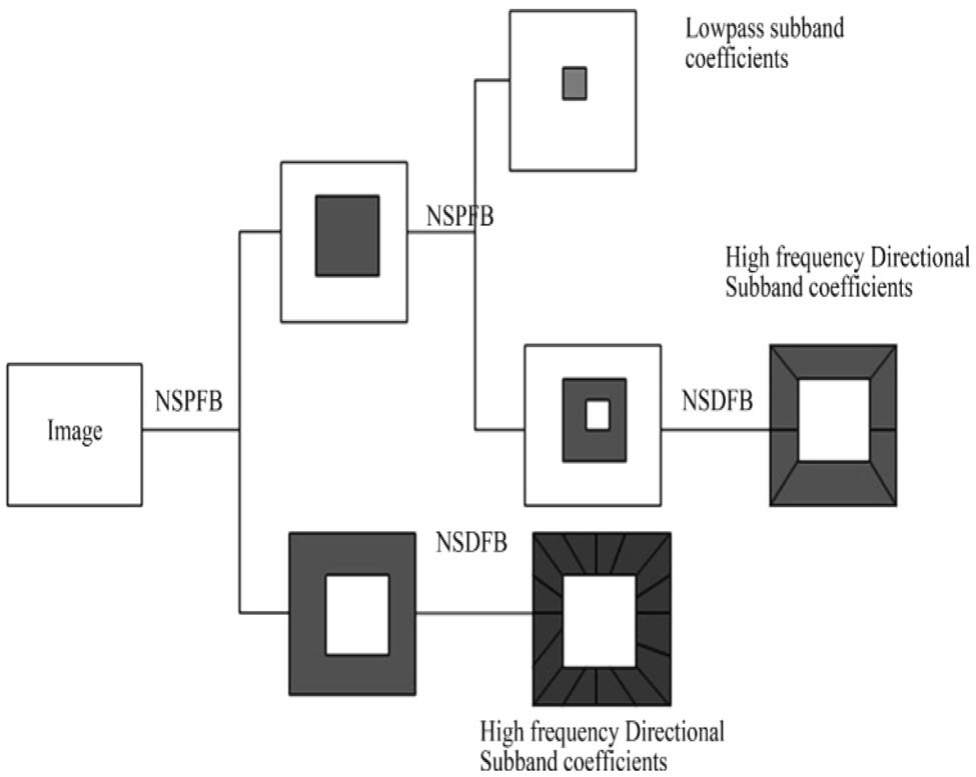
The NSCT image decomposition process [21]

**Fig. 11:**
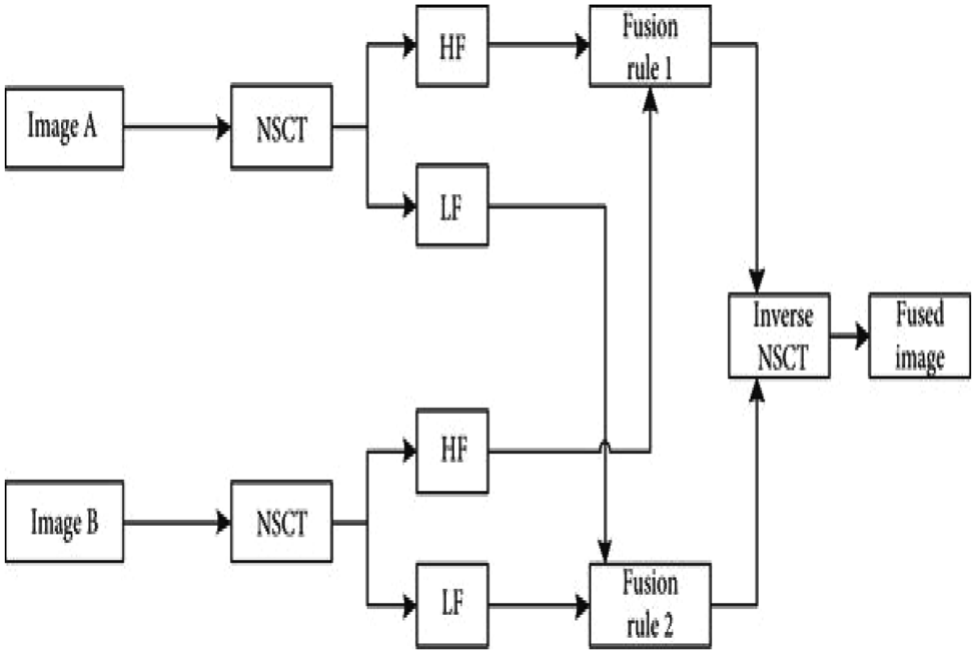
The NSCT based fusion process [5]

The contourlet transform technique, which is helpful in image processing, is the basis of NSCT. A shift variation problem with the contourlet transform is caused by down- and up-sampling [2, 21]. The NSCT method is a multi-scale, shift-invariant, and multi-directional transform. This method ensures the multi-directional and multi-scale by applying the Non-Subsampled Directional Filter Bank (NSDFB) and the Non-Subsampled Pyramid Filter Bank (NSPFB) [2, 21]. Figure 10 illustrates the NSCT decomposition process. Figure 11 illustrates the basic NSCT fusion process.

Das et al. [55] presented a MIF method using the PCNN in the NSCT domain. The NSCT decomposes the medical images, then the PCNN and the max selection methods merge the high- and low-frequency subbands. Use the inverse of the NSCT method to create the output image. Comparison results demonstrate that the presented method correctly combines medical images.

Bhatnagar et al. [56] proposed a novel fusion method using the NSCT method for fusing medical images. The NSCT transformed the original images, resulting in low- and high-frequency subbands. The high- and low-frequency subbands are merged using the directive contrast and the normalized Shannon entropy methods. These methods preserve more functional and spatial information from the original images. Comparative studies demonstrate that the proposed fusion method accurately fuses medical images.

Tian et al. [57] proposed a novel fusion method for combining medical images using the NSCT and improved pulse-coupled neural network (IPCNN) method. The proposed MIF method performs better than the other methods.

Zhu et al. [2] presented a mix of local Laplacian energy and phase congruency methods to fuse medical images in the NSCT. This method is performed in three steps. Use the NSCT method to decompose the source images. The phase congruency method merges the highpass subbands, whereas the lowpass subbands are combined using the local Laplacian energy method. The final image is generated using the inverse of the NSCT method. The presented method merges CT, MRI, and SPECT images with low computational time and high accuracy. On the other hand, it is not good to fuse the MRI and PET images.

Sa et al. [21] proposed an MMIF method using the NSCT and PCNN methods. The NSCT decomposes the input images into low- and high-frequency coefficients. The PCNN merges both the high- and low-frequency coefficients. The proposed method used six collections of medical images in different modalities: CT / MRI, MR-T1 / MR-T2, MR-T2 / SPECT, and MR-T1 / PET. The proposed method’s performance is evaluated using these measures: peak signal-to-noise ratio (PSNR), weighted edge information (Q${}^{AB/F}$), average gradient (AG), nonlinear correlation information entropy (Q${}_{ncie}$), SD, MI, and EN. The experimental results show that the proposed method outperforms the compared fusion methods. The benefits of the proposed method achieve promising results that help doctors detect the accurate position of the disease and treat patients.

Lv et al. [58] proposed a Rybak neural network with two channels to perform multi-focus image fusion in the NSCT domain. Unlike PCNN, this technique explicitly models a neural network while also processing both source images, which ensures consistent spatial checks throughout. This results in higher complexity than PCNN. The technique has been verified using Lytro, MFFW, and MFI-WHU datasets, which include grayscale images.

#### PCNN fusion method

**Fig. 12:**
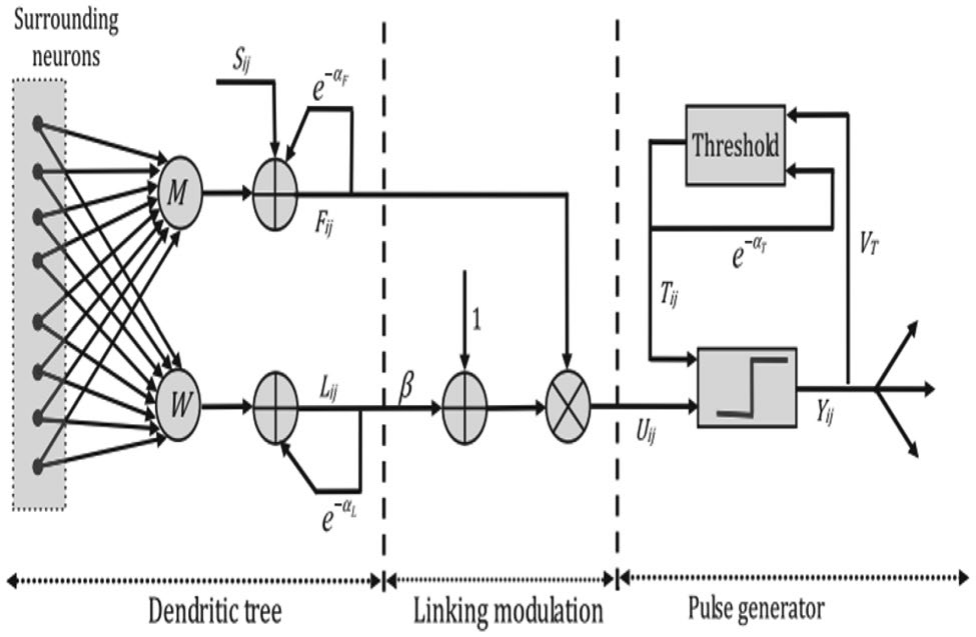
Architecture of the PCNN model [21]

PCNN is the tertiary generation of an artificial neural network technique developed by Eckhorn et al. in 1990 [59, 60]. It is used in many areas, including image processing and image fusion, without a training process, especially for medical images [21]. The PCNN architecture is depicted in Fig. 12. This architecture involves three parts: a dendritic tree receives inputs, modulation linking receives an external stimulus, and a pulse generator. The PCNN model can be mathematically illustrated by the equations below [21].

1 $$f_{ij}(n)=e^{-\alpha_{f}}f_{ij}(n-1)+v_{f}\sum_{kl}m_{ijkl}y_{kl}(n-1)+s_{ij}$$

2 $$l_{ij}(n)=e^{-\alpha_{l}}f_{ij}(n-1)+v_{l}\sum_{kl}w_{ijkl}y_{kl}(n-1)$$

3 $$u_{ij}(n)=f_{ij}(n)(1+\beta l_{ij}(n))$$

4 $$y_{ij}(n)=\begin{cases}1, \ u_{ij}(n) > h_{ij}(n-1)\\0, \ otherwise\end{cases}$$

5 $$h_{ij}(n)=e^{-\alpha_{h}}h_{ij}(n-1)+v_{h}y_{ij}(n-1)$$

where the PCNN input channels are $l_{ij}$(linking channel) and $f_{ij}$(feeding channel) in the $i$ and $j$ position. The external stimulus is denoted by $s_{ij}$, the local matrix are defined by $m_{ijkl}$ and $w_{ijkl}$. The $y_{ij}$ is represent the neuron’s output. The time constants are defined by $\alpha_{f},$ $\alpha_{l}$ and $\alpha_{h}$. The linking coefficient is $\beta$. The voltage are defined using $v_{f}$, $v_{l}$ and $v_{h}$.

Xia et al. [60] presented a fusion method that combines the SR and PCNN methods in the NSCT transform to merge medical images. The NSCT decomposes the original medical images to obtain high- and low-frequency subbands. The PCNN and SR methods combine high- and low-frequency subbands, respectively. Apply the NSCT reconstruction to produce the fused image. The result was tested using the main fusion measures: MI, EN, SD, SF, AG, SSIM, and edge information delivery. The experimental results show that the fused image has higher contrast and performance than the other main fusion methods. The presented fusion method solves the defect of the NSCT method in the non-sparse low-frequency subbands.

Huang et al. [61] used the PCNN and the shuffled frog leaping algorithm (SFLA) for fusing the CT and SPECT images. First, the NSCT and IHS decomposed these images. The PCNN and SFLA base fusion methods combine low- and high-frequency images. The reverse of both the NSCT and IHS methods reconstructs the final image. The SFLA optimizes the PCNN parameters. The evaluation of the applied fusion method includes four fusion metrics. According to the results, the used fusion method outperforms others in keeping source image information.

Li et al. [62] presented a mix of the PCNN and NSCT methods to merge the medical images. NSCT decomposed the input images to get high and low-frequency bands. A PCNN combines the low-frequency bands, whereas the high-frequency bands are fused by the WSEML and the guided image filtering (GIF) method. The presented technique merges medical images with high performance.

Sa et al. [63] proposed the MMIF method using the PA-PCNN and the NSCT methods. The NSCT decomposes original images into high- and low-frequency coefficients. The PA-PCNN combines those coefficients. The proposed method used eight collections of medical images in different modalities: CT/MR, MR-T1/MR-T2, MRI/PET, and MRI/SPECT. The proposed method’s performance is evaluated using these measures: EN, MI, Q${}^{AB/F}$, Q${}_{ncie}$, and AG. Results obtained that the proposed method gains high performance when compared with the five MIF methods.

Lv et al. [64] presented an adaptive parameter-controlled PCNN (AGPCNN) with fractal dimension analysis in the NSCT domain. The advantages include automated parameter optimization, which eliminates manual configuration, integrity checking, which reduces artifacts, and NSCT, which provides shift-invariant multidirectional decomposition. The disadvantages include high computational time and insufficient validation for medical images. The approach was tested on the Lytro and MFI-WHU datasets.

Li et al. [65] presented a Fractal dimension analysis that was combined with a parameter-adaptive unit-linking dual-channel PCNN in the Curvelet transform domain. Advantages include suitability for curved edges, ease of computation using unit-linking PCNN, and automatic parameter changes. The disadvantages include long computing times and limited medical image validation. This work utilized Lytro, MFFW, and MFI-WHU datasets, which comprised both grayscale and color photographs.

Lv et al. [66] suggested combining PAPCNN with fractal dimension analysis in the NSST domain to fuse multi-focus images. Advantages of NSST include superior directional sensitivity compared to NSCT and automated adaptation of PCNN settings. Disadvantages include excessive computing time and poor support for medical images. This work utilized Lytrodatasets for grayscale and color images.

### Fuzzy fusion method

For more complex computations, the fuzzy transform is primarily used to convert a discrete or continuous function into a finite vector. It is used in various areas like removing noise, image processing, image classification, image compression, pattern recognition, image segmentation, and image fusion [67]. The fuzzy transform removes noise while retaining image properties with little computational time [68].

Javed et al. [69] presented a fuzzy logic method and local features for fusing PET and MRI modalities. Results declare that the presented method achieves high results according to the quantitative and visual analyses.

Manchanda et al. [68] fused medical images using the fuzzy transform. The proposed method uses eight medical datasets. The results of objective and subjective evaluations show that this fusion method is of higher quality than the other methods compared. The advantage of the proposed method is that it produces images of high quality with more clarity and without any artifacts.

Yang et al. [70] proposed a fuzzy logic and structural patch decomposition (SPD) method to combine various medical images. The SPD method yields two salient features. The incomplete and supplemental fusion maps are extracted from the salient features. These maps are merged by the fuzzy logic method. The weighted average obtains the fused image. Results show that the proposed fusion method enhances the visual effects of the original image while retaining image details.

Li et al. [71] proposed an MMIF method using a mix of the NSCT and fuzzy entropy methods. First, NSCT decomposes the input images to obtain the low- and high-frequency coefficients. The fuzzy entropy and regional energy fuse the low-and high-frequency components, respectively. Finally, the inverse of the NSCT method obtains the fused images and retains image details.

### Deep learning-based medical image fusion methods

#### Auto-encoder-methods

A lightweight auto-encoder-based fusion architecture with dense blocks is proposed [72], where each layer’s output connects to every other layer to enhance feature reuse and data flow. The encoding network incorporates convolutional layers and dense blocks, utilizing two fusion algorithms to merge features within the latent space. The architecture is applicable to modalities such as infrared and visible images. Key advantages include a basic encoder-decoder structure, rapid computational runtime, and minimal training data requirements. However, limitations include insufficient attention to texture information and the inability to automatically learn optimal fusion procedures.

In [73] the RFN-Nest advances upon traditional autoencoder methods by incorporating residual fusion networks and nest-connected decoders, which facilitate the preservation of both local and global information through multi-level feature reuse. The nest connections support information flow across varying spatial scales, thereby enhancing detail retention. The model is applicable to modalities such as infrared, visible, and medical images. Key advantages include improved gradient flow during training due to residual fusion networks, maintenance of multi-level features by nest-connected decoders, and superior detail retention compared to DenseFuse. significantly exceeding DenseFuse), complex training process render it unsuitable for resource-constrained devices or real-time, applications.

#### CNN-based method

**Fig. 13:**
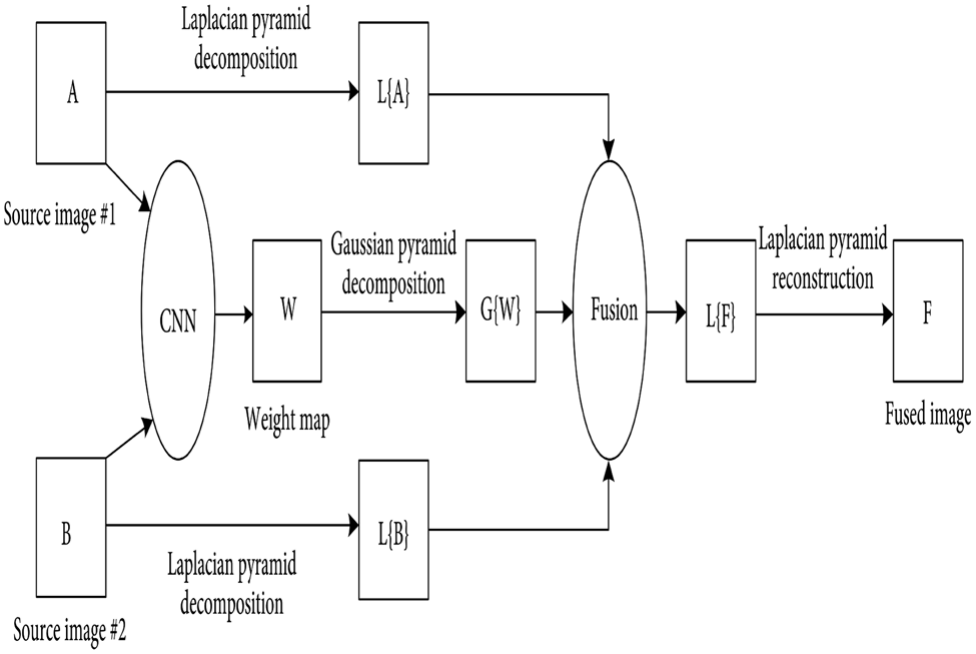
CNN based image fusion [5]

CNN is considered one of the most common deep learning methods. It’s trying to find a way to represent hierarchical features for images at various layers [74]. Deep learning is used in different fields, such as medical image segmentation, image classification, image recognition, and medical image registration. It includes convolutional, max pooling, and fully connected layers [4, 74]. The feature extraction component of the system is often seen as the convolutional and max-pooling layers, while the classification part is considered the fully connected layers. Figure 13 describes the process of using CNN for image fusion.

Liu et al. [17] introduced CNN to fusing medical images. The introduced fusion method includes four phases: generation of the weight map, decomposition, coefficient fusion, and reconstruction. First, CNN computes the weight map. Second, an LP method decomposes each input image. After that, the low- and high coefficients are merged. Finally, use the inverse of the LP method to create the resulting image. The introduced method uses a variety of medical image modalities using MATLAB. The results suggest that the utilized technique produces higher quality results than other standard fusion methods.

Hermessi et al. [20] presented a CNN-based fusion in the NSST domain. The NSST decomposes the registered images into low- and high-frequency subbands. Then, the CNN feature extraction merges high-frequency coefficients, where the local energy fuses low-frequency coefficients. The fused image is produced via the inverse NSST. The presented technique is tested on CT and MR images using MATLAB R2016a. Use evaluation metrics like MI, En, SD, Image Quality Index (IQI), Chen & Blum metric (CB), SSIM, and SF. Results show that the presented technique achieved high performance compared with others with low computational time.

Xia et al. [75] suggested an MMIF method that includes the properties of deep convolutional neural networks (DCNN) and MST. Results reveal that the suggested method obtains better results using different quantitative metrics when compared to other fusion methods. The main advantage of using this method is receiving high-quality fusion results at a low computational time. On the other hand, the fusion rule needs to be enhanced to obtain the adaptive MMIF method.

Ding et al. [4] applied a new method for brain image fusion based on CNN in NSST. CNN calculates the weight map. First, the NSST decomposed the initial weight into high and low-frequency coefficients. The activity level measurement fused the first low-frequency coefficients. The CNN strategy for the weight map merges the second low-frequency coefficients. The initial weight’s high-frequency components combine the high-frequency coefficients. Finally, the inverse NSST regenerates the final result. The pairs of CT, MRI, PET, and SPECT images evaluated the performance of the presented technique. The results demonstrate that the applied method achieved high performance in fusing medical images and preserving all meaningful information.

Wang et al. [76] introduced the MMIF method based on the CNN and NSCT methods. To begin with, the NSCT deconstructs the source images. Second, the decision map and the perceptual high-frequency CNN (PHF-CNN) merge the low-and high-frequency subbands. The NSCT then reconstructs the final image. The results indicate that the introduced strategy is acceptable for combining medical images.

Li et al. [77] presented supervised learning and a CNN-based method for fusing medical images. It also extracts the most relevant characteristics. The fusion method includes two major phases: training and fusion testing. First, use large datasets and preprocess these datasets. Second, perform the fusion method. The experiment uses these images: MRI, CT, and SPECT. Results indicate that the presented method improves the fusion outcome and saves time, especially for medical images.

El-Shafai et al. [78] suggested combining medical photos using the CNN approach. Focus detection is the first step in this process, followed by initial segmentation, consistency verification, fusion, and the third stage. Using the raw photos, the first step attempts to generate a focus map. Applying focus map segmentation with a 0.5 threshold to produce a binary map is the second stage’s output. We eliminated the noise and applied the guided image filter for the binary map segmentation during the consistency verification stage. The fused image was obtained in the last stage using the weighted average approach. CT and MR scans were used in the experiment. The outcomes show how beneficial the suggested fusion procedure is for diagnostic applications.

Li et al. [79] explored the use of coupled neural P systems and fractal dimension analysis in the NSCT domain to merge multi-focus images. Advantages include the ability to communicate between two systems and analyze data in parallel. Disadvantages include complexity, difficulty in implementation, and poor support for medical images. Grayscale images were processed using the Lytro, MFI-WHU, and MFFW databases.

Zhang et al. [80] presented an image fusion method that uses a CNN and a siamese-based architecture with shared weights. The network extracts features at different scales and combines them using a maximum fusion approach, then reconstructs the final image. This method works with multi-focus, infrared-visible, and medical images. Its main advantages are fast inference time and a flexible framework for many fusion tasks. However, it uses a large dataset and may not work well with all modality combinations.

In [81], U2Fusion is an unsupervised end-to-end image fusion network that can handle different fusion challenges, such as medical, multi-focus, and multi-exposure cases. It uses feature extraction and information assessment to decide which parts of the source images are important and keeps that information. The main advantages are that it works for different fusion tasks, learns without supervision, adapts to preserve information, and performs well on multi-image fusion. However, it has a complex loss function, runs slower than IFCNN, and might not always give the best results for every task.

#### Generative adversarial network (GAN)-based methods

FusionGAN in [82] is the generator that creates a fused image incorporating both modalities, while the discriminator requests the generator to preserve more information from the visible image. Together, adversarial training contributes to visually accurate outcomes. In this process, images might be infrared or visible. There are several advantages, such as a visually clear image and efficient management of modality imbalance. However, some disadvantages exist. For example, there is a failure risk. Additionally, infrared brightness information might be ignored, two adversarial losses are difficult to balance, inference is delayed, and there is an increase in artifacts.

DDcGAN [83] suggests a dual-discriminator conditional GAN, with one discriminator focusing on global structure and the other on local details. This dual-discriminator technique enhances detail retention and training consistency. Modalities include infrared, visible, and medical images. Advantages include capturing both global and local accuracy, detail retention, increasing training durability, performing well on medical image fusion tasks, and handling multi-resolution inputs. Disadvantages are a higher computational cost than FusionGAN; longer training time.

Different MIF techniques have been proposed in this section for a comprehensive overview of earlier and recent approaches [1, 6, 22, 27, 28, 63, 84–86]. Table 3 presents an overview and discussion of various MIF techniques, highlighting these techniques, image modality, domains, benefits, and limitations of each method.

**Table 3:** Comparison of various MMIF methods in several research papers [63]

Related work	Year	Image Modality	Method	Domain	Advantages	Disadvantages
He et al. [31]	2010	MRI/PET	PCA and IHS transform	Spatial Domain	Protect spatial information	Cause color distortion
Krishn et al. [32]	2015	CT/MR-T1				
		CT/MR-PD	PCA and ridgelet transform	Spatial Domain	protects edges and contrast information	uses specific image modality
Himanshi et al. [33]	2016	CT/MR-T1	PCA and Maximum			
		CT/MR-PD	Selection Rule	Curvelet Domain	protects edges and	uses specific image modality
					curved shapes	
J et al. [34]	2018		cascaded PCA and		improves exact edge details	
		MRI/CT	shift invariant	Wavelet Domain	with minimizing distortions	uses specific image modality
			wavelet transforms			
Bashir et al. [35]	2019	CT and MRI			provides higher performance	acheives lower performance
		X-ray and MRI	SWT and PCA	Wavelet Domain	while images with variety	when the input images are distinct
		MRI and SPECT			brightness levels and contrast	cannot deal with
						multi-focus and multi-view images
Ghandour et al. [36]	2024	MRI/CT				
		MRI/SPECT	PCANET and the nuclear norm	PCA	protect image details	
		MRI/PET				high computational time
Daneshvar et al. [37]	2010	PET/MRI			reduces color distortion and	uses specific image modality
			IHS and RIM	Spatial Domain	does not need resampling process	
Haddadpour et al. [87]	2017	MRI and PET	IHS and 2DHT	Spatial Domain	preserves the spatial and spectral	uses specific image modality
					features from the source images	
Chen [88]	2017	MRI and PET	IHS and	Spatial Domain	prtoects image details	Lack of any preprocessing steps
			Log-Gabor transform			dealing with unregistered images
Dilmaghani et al. [89]	2017	MRI and PET	IHS and BEMD	Spatial Domain	prtoects spectral information	Lower entropy value than wavelet
Sahu et al. [39]	2014	CT and MRI	LP and DCT	LP Domain	contains moreedge information	uses specific image modality
Li et al. [40]	2019	CT/MR				
		MR-T1/MR-T2	SR and SML	LP domain	preserves functional information	not suitable for
		PET/MR			with high contrast	fusing SPECT/MR images
		SPECT/MR				
Liu et al. [41]	2020	CT and MRI	LP and CSR	LP domain	extacts texture information	use specific image modalitiy
Li et al. [42]	2020	MRI-SPECT	LRD framework	LP domain	high performance	faces color distorations
		MRI-PET				and blurring problem
Wang et al. [16]	2020				protects edges and	needs some update to be
		CT-MRI	LP and ASR	LP domain	structure information	sutible for fusing
						other image modalaities
Li et al. [43]	2024	Infrared-visible				
	Multi-focus		SR and guided filter	LP domain	protects edges	not medical-specific
					no training	
Teng et al. [44]	2010	CT/MRI	absolute value			
		PET/MRI	maximization and	DWT Domain	retains image details	Not good for color images
			weighted averaging			
Bhavana et al. [45]	2015	MRI and PET	DWT	DWT Domain	retains structural information	loss edge information
					without colordistortion	
Soundrapandiyan et al. [46]	2017					not suitable for fusing
		CT/MRI	IFS and DWT	DWT Domain	produces high contrast image	color images
Ganasala et al. [48]	2014	CT-MRI	sum of variations and	NSST Domain	protect bone and	cause color distortion
		SPECT-MR	activity level parameters		anatomical details with	loss edge information
					good contrast	
Jin et al. [47]	2018	CT, MRI,			protect functional and	loss some
		and PET	ICM and	NSST	structural information of	edges information
			S-PCNN		the original images	
Yin et al. [49]	2019	CT/MRI				
		MR-T1/MR-T2	PA-PCNN	NSST	high performance	time consuming
		MRI/PET				
		MRI/SPECT				
Gai et al. [50]	2019	MR-T1/CT				
		MR-T1/MR-PD				
		MR-T1/MR-T2	ISR and	NSST	high performance	distorted result
		MR-T2/MR-Gad	EP-PCNN			and loss some details
		MRI/SPECT				of the source images
		MRI/PET				
Vanitha et al. [51]	2021	CT/MR,				
		MR-T1/MR-T2	SF-PAPCNN	NSST	preserving edge	not suitable for
		MRI/PET			information and	merging CT, MRI,
		MRI/SPECT			structural details	and SPECT image
Mei et al. [52]	2022	CT/MRI	PCNN		achieves better	uses only CT
			and SDG	NSST	results and low	and MRI images
					computational time	
Vanitha et al. [53]	2022	CT/MR,				
		MR-T1/MR-T2	PA-PCNN and	NSST	used in disease	some edges cannot
		MRI/PET	energy attribute		treatment	be detected
		MRI/SPECT				
Guo et al. [54]	2023	CT/MRI	MI and CSR			
		MRI/SPECT		NSST	high contrast images	consume more time
Das et al. [55]	2012	CT/MRI				
		MR-T1/MRA	modified SF			use specific
		CT/MR-GAD	and PCNN	NSCT	high spatial	images modalilities
		MR-T1/MR-T2			resolution images	and still low
		CT/MR-PD				quality images
Bhatnagar et al. [56]	2015	CT/MR	directive contrast			
		MR-T1/MR-T2	and normalized	NSCT	protect image details	consume more time
			Shannon entropy			
Tian et al. [57]	2016	MRI/CT				
		MRI/SPECT	NSCT and	NSCT	protect edge details	loss some dteails from
		PET/MR-T1	IPCNN		with no distortions	the original images
Zhu et al. [2]	2019	CT/MRI	phase congruency		high-performance and	blurred image and
		MRI/PET	and local Laplacian	NSCT	low computational time	not suitable for
		SPECT/MRI	energy			fusing PET-MRI images
Sa et al. [21]	2023	CT/MR-T2				
		CT/MR-PD			achieve promising results	
		CT/MR-Gad	PCNN	NSCT	to detect the	time cosuming
		MR-T1/MR-T2	in NSCT domain		accurate position	
		MRI/PET			of the disease	
		MRI/SPECT			and treat patients	
Lv et al. [64]	2026	Multi-focus	AGPCNN, fractal dimension	NSCT	automated parameter optimization	High complexity
		CT-MRI			reduces artifacts	slow
		PET-MRI			shift-invariant	limited medical validation
Li et al. [65]	2025	Multi-focus	parameter-adaptive unit-linking dual channel PCNN	Curvelet	Curved edges	Very high cost
					automatic parameter changes	limited medical validation
Lv et al. [58]	2025	Multi-focus	Adual-channel Rybak NN	NSCT	automated adaption	High complexity
					parallel processing	
Lv et al. [66]	2023	Multi-focus	PAPCNN and fractal dimension	NSST	automated adaption	High computational time
					superior directional sensitivity	limited medical validation
Javed et al. [69]	2014	MRI/PET	Fuzzy transform	Fuzzy	protect information of	loss some
					both source images	spectral information
Manchanda et al. [68]	2018	MRI/CT			produces high clarity images	
		MRI-T1 /MRI-T2			with sharp and	
		CT/PET	Fuzzy transform	Fuzzy	smooth edges	high cost
		MRI/SPECT			free of artifacts	
		MRI/PET				
Yang et al. [70]	2019	MR-GAD / MR-T2				
		CT / MR-T2				
		MR-T1 / MR-T2	fuzzy logic and SPD	Fuzzy	protect information of both	loss some spectral
		MR-T1 / MRA			source images	information
		CT / MRI				
Xia et al. [60]	2018	CT/MRI				
		MR-PD/MR-T1				
		MR-PD/MR-T2			preseves edge information and	lose some information
		MR-T1/MR-T2	SR and PCNN	NSCT	solves the defect of the	from the original images
		MR-PD/PET			NSCT method	
		MR-T1/PET				
		MR-T2/PET				
Huang et al. [61]	2019	SPECT and CT	SFLA and PCNN	IHS and NSCT	preseves image information	The efficiency need
						to be enhanced
Li et al. [62]	2021	CT/MRI				
		MR-T1/MR-T2	PCNN, GIf,	NSCT	enhances the contrast	
		MRI/PET	and WSEML		of the result images	high computational
		MRI/SPECT				time
Sa et al. [63]	2023	CT/MR			preseves edge information	It is not good for
		MR-T1/MR-T2	NSCT and PAPCNN	NSCT	without noise and	fusing MR-T1 and
		MRI/PET			preseves image contrast	MR-T2 images
		MRI/SPECT				
Li & Wu [72]	2019	Infrared-visible			simple	
			DenseFuse	Auto-encoder	effective computational runtime	Handcrafted fusion rules
Li et al. [73]	2020	Infrared-visible	Residual fusion and nest connections	Autoencoder	superior detail retention	complex training
Liu et al. [17]	2017	CT/MR			preseves energy	
		MR-Gad/MR-T1	CNN	LP	and image details	high computational
		MR-T1/MR-T2			without visual artifacts	time
		MR-T2/SPECT				
Hermessi et al. [20]	2018	CT/MRI	CNN	NSST	preseves image details	high computational
						time
Xia et al. [75]	2019	CT/MRI	largest local variance		high-quality image	the fusion rule
		CT/PET	and local energy	DCNN	with low computational time	need to be enhanced
Ding et al. [4]	2020	CT/MRI	The activity level			need to be enhanced
		CT/PET	measurement, the weight	NSST	high performance	to solve the
		CT/SPECT	map, the initial weight,			CNN weight problem
			and the CNN			
Wang et al. [76]	2021	CT/MR-GAD				
		CT/MR-T1				high comutational
		CT/MR-T2	PHF-CNN	NSCT	enhances the quality	time and cannot
		MR-T1/MR-T2			of result images	fuse color images
		MR-T1/MR-GAD				
Li et al. [77]	2021	CT/MRI	supervised learning		high performance and	good only for
		MRI/SPECT	and a CNN	CNN	low computational time	medical images
El-Shafai et al. [78]	2023	CT/MR	CNN	CNN	useful for real time	need to enhance to
					medical applications	apply vision transformers
Li et al. [79]	2024	multi-focus	coupled neural P systems	NSCT	Bio-inspired	difficult implementation
			and fractal dimension			no medical validation
Zhang et al. [80]	2020	Multi-focus, IR	Siamese CNN with			
		and Medical	shared weights	CNN	Fastest	Requires large dataset
Xu et al. [81]	2021	Multi-focus				may lower contrast
		and Medical	Unified unsupervised CNN	CNN	multi-task	complex and slower
Ma et al. [82]	2019	Infrared + Visible				Higher complexity
			GAN	GAN	better details	need more memory
						Can introduce artifacts
Ma et al. [83]	2020	Multi-resolution				
		IR-Visible				Computationally heavily
			Dual-Discriminator		Handles resolution differences well	limited medical validation
		Multi-focus	Conditional GAN	GAN		

## Datasets

In the medical area, MIF datasets play a crucial role in enhancing and evaluating image fusion techniques by merging information from different medical imaging modalities, like MRI, CT, PET, and SPECT to create more detailed and informative images that assist doctors in diagnosing and treating diseases. MIF datasets typically consist of composed modalities and high-resolution images without artifacts, allowing for efficient fusion and estimation. These qualities make MIF datasets suitable for a wide range of clinical applications. There are various medical image fusion datasets, the most common datasets in this area are discussed below:

- **The Harvard Whole Brain Atlas** is a widely utilized MIF dataset in numerous studies. This comprehensive dataset encompasses various modalities for abnormal and normal brain images, including MRI, CT, SPECT, and PET. Derived from real patients, the images meticulously document their diseases and conditions, rendering them an invaluable resource for tumor studies. This dataset can be accessed using this link; https://www.med.harvard.edu/aanlib/.
- **The Cancer Imaging Archive (TCIA)** dataset includes multimodal brain images of cancer patients, encompassing MRI, CT, and PET image modalities, and is utilized for cancer detection. The researchers access this dataset by https://www.cancerimagingarchive.net/.
- **Alzheimer’s Disease Neuroimaging Initiative (ADNI)**: This dataset merges MRI and PET brain images for Alzheimer’s disease detection. The goal is to preserve both structural and functional brain information. The website for using this dataset is https://adni.loni.usc.edu/.
- **Open Access Series of Imaging Studies (OASIS)** dataset uses different modalities for brain MRI scans to detect Alzheimers disease. It is accessed using https://sites.wustl.edu/oasisbrains/.
- **Information eXtraction from Images (IXI)** dataset contains various modalities of MRI scans like T1-weighted, T2-weighted, and Proton Density(PD) for brain images. It is useful for brain image analysis, registration, and fusion tasks. The website for this database is https://brain-development.org/ixi-dataset/.
- **Brain Tumor Segmentation Challenge (BRATS)** uses various modalities for brain MRI scans. It focused on image segmentation and tumor detection. This is accessed by http://braintumorsegmentation.org/.

## Quality measures

Image fusion aims to combine two input images from different modalities into one image that protects more information from the input images. Various metrics are used to evaluate the quality of the fused image. Fusion metrics are essential to ensuring the effectiveness of MIF techniques. These metrics are classified into objective and subjective metrics. The subjective metrics rely on visual estimation and are used to compare the source images with the merged image. These metrics, also known as qualitative metrics, evaluate the quality of the final fused images and depend on human observation. These metrics include image size, image details, clarity, sharpness, contrast, and color. All of these metrics are used when evaluating MIF techniques [22].

Objective metrics, also known as quantitative metrics, are derived from mathematical operations without any human intervention. There are several objective metrics include:

- **Mutual Information (MI):** measures the information from both the fused image and the source images. Greater mutual information signifies superior fusion [21]. MI is given by:
  6 $$MI=MI_{AF}+MI_{BF}$$
  where $A$, $B$ are the source images and the fused image is noted by $F$. The high MI value means the high performance fused image. $MI_{AF}$ is the mutual information between the images $A$ and $F$. $p_{A,F}(m,n)$ is the joint probability of the images $A$ and $F$.
  7 $$MI_{AF}=\sum_{m,n}p_{A,F}(m,n)\log_{2}\left[\frac{p_{A,F}(m,n)}{p_{A}(m)p_{F}(n)}\right]$$
- **Structural Similarity Index Measure (SSIM)** evaluates the structure similarity between both the oraginal image and the fused image that considering contrast, luminance, and structure in account. The range value for this metric from −1 to 1, where the 1 is the high structure similarity.
  8 $$\mathrm{SSIM}(x, y) = \frac{(2\mu_x \mu_y + C_1)(2\sigma_{xy} + C_2)}{(\mu_x^2 + \mu_y^2 + C_1)(\sigma_x^2 + \sigma_y^2 + C_2)}$$
  Where $\mu_x$, $\mu_y$ are the mean of $x$ and $y$ images. $\sigma_{xy}$ is standars deviation between $x$ and $y$. $\sigma_x^2$, $\sigma_y^2$ are the variances of images $x$ and $y$. $C_1$ and $C_2$ are constants.
- **Average Gradient (AG):** the gradient Information of the combined image is evaluated by this metric. It also measures the texture detail such as sharpness and clarity of the fused image [63, 84, 90]. High AG value means the fused image with high performance. The AG metric is given by this equation
  9 $$AG = \sum\limits_{m = 1}^M {\sum\limits_{n = 1}^N {\sqrt {\tfrac{{\left( \begin{subarray}{l} {(F(m,n) - F(m + 1,n))^2} \\ \quad \quad \quad + {(F(m,n) - F(m,n + 1))^2}/2 \end{subarray} \right)}}{{MN}}} } }$$
- **Spatial Frequency (SF):** evaluates the sharpness and details of image. The higher SF value is more sharper image. SF is combined both RF and CF. The SF metric is combined both row frequency (RF) and column frequency (CF) given by these equation [27]:
  10 $$\mathrm{RF} = \sqrt{\frac{1}{m(n-1)} \sum_{i=1}^{m} \sum_{j=1}^{n-1} \left( I(i, j+1) - I(i, j) \right)^2}$$
  11 $$\mathrm{CF} = \sqrt{\frac{1}{(m-1)n} \sum_{i=1}^{m-1} \sum_{j=1}^{n} \left( I(i+1, j) - I(i, j) \right)^2}$$
  12 $$\mathrm{SF} = \sqrt{\mathrm{RF}^2 + \mathrm{CF}^2}$$
  where $I(i, j)$ is a pixel value at position $i$ and $j$.
- **Mean Squared Error (MSE)** is the average squared difference between the original image $x(i, j)$ and the fused image $\bar{x}(i, j)$. Low MSE value indicates perfect fusion performance. It is given by the following equation:
  13 $$\mathrm{MSE} = \frac{1}{MN} \sum_{i=1}^{M} \sum_{j=1}^{N} \left( x(i, j) - \bar{x}(i, j) \right)^2$$
- **Weighted edge information** $$($$Q$${}^{AB/F})$$: Total information transferred and edge intensity information from source images to the fused image, which is given as [21, 63]:
  14 $${Q^{AB/F}} = \tfrac{{\sum\limits_{m = 1}^M {\sum\limits_{n = 1}^N {\left( \begin{subarray}{l} {Q^{AF}}(m,n){W_A}(m,n) \\ \quad \quad \quad + {Q^{BF}}(m,n){W_B}(m,n) \end{subarray} \right)} } }}{{\sum\limits_{m = 1}^M {\sum\limits_{n = 1}^N {({W_A}(} } m,n) + {W_B}(m,n))}}$$
  where the preservation factors of the edge information are denoted by $Q^{AF}$ and $Q^{BF}$, and the weighted items represented by both $W_{A}$, $W_{B}$. The $Q^{AB/F}$ range is between 0 and 1. Where the 1 value indicates the fused image is preserved more edge information.
- **Normalized Cross-Correlation (NCC)** evaluates the similiarity and correlation between both the source images and the fused image.
  15 $${\mathrm{NCC}} = \frac{{\sum\limits_{i = 1}^m {\sum\limits_{j = 1}^n {({I_f}(} } i,j) - {{\bar I}_f})({I_r}(i,j) - {{\bar I}_r})}}{{\sqrt {\sum\limits_{i = 1}^m {\sum\limits_{j = 1}^n {({I_f}(} } i,j) - {{\bar I}_f}{)^2}} \sqrt {\sum\limits_{i = 1}^m {\sum\limits_{j = 1}^n {({I_r}(} } i,j) - {{\bar I}_r}{)^2}} }}$$
  where $I_f(i,j)$ and $I_r(i,j)$ are pixel intensity of the $I_f$ and $I_r$ images at $i$ and $j$ positions. $m$ and $n$ represent row and column in the image. $\bar{I}_f$ and $\bar{I}_r$ denote the mean intensity of the fused and reference images. The range of NCC values from −1 to 1, where 1 is perfect correlation, 0 is no correlation, and −1 is perfect not correlation.
- evaluates the average difference between the original image and the fused image. It given by this equation:
  16 $$\mathrm{AD} = \frac{1}{mn} \sum_{i=1}^{m} \sum_{j=1}^{n} |I_f(i,j) - I_r(i,j)|$$
- **Entropy (EN)** measures the amount of information in the final fused image. A high EN value indicates a highquality image. It’s defined as follows:
  17 $$EN=-\sum_{l=0}^{L-1}p_{l}\log_{2}p_{l}$$
  where $p_{l}$ is the probability of $l$ grey levels and $L$ is the number of grey levels in an image [21, 57, 63, 84].
- **Peak Signal to Noise Ratio (PSNR):** one of the main evaluation metrics to measure the quality of the fused image. The high PSNR values represent high quality images [21] and is given by this equation:
  18 $$PSNR=10\log_{10}\left[\left(255\right)^{2}/MSE\right]$$
- **The standard deviation (SD)** of the fused image contrast is calculated based on the spread of the image information. A high SD value is a high-contrast image [21, 63]. The SD formula is represented by this equation:
  19 $$SD = \sqrt {\frac{{\sum\limits_{m = 1}^M {\sum\limits_{n = 1}^N {{{\left( {F(m,n) - \mu } \right)}^2}} } }}{{MN}}}$$
  where $MN$ is the size of the input image $F(m,n)$ and $\mu$ is the mean of the fused image The $\mu$ can be defined as follows:
  20 $$\mu = \frac{{\sum\limits_{m = 1}^M {\sum\limits_{n = 1}^N F } (m,n)}}{{MN}}$$

All of the perivous metrics in several research paper are discussed and summarized in Table 4.

**Table 4:** Comparative study of various related work

Related work and Year	Method	Image Modality	Organ	Dataset	MI	SSIM	AG	SF	MSE	Q${}^{AB/F}$	NCC	AD	EN	PSNR	SD
He et al. [31] 2010	PCA and IHS transform	MRI/PET	Brain	AANLIB	2.9546										
Krishn et al. [32] 2015	PCA and ridgelet transform	CT/MR-T1	Brain			0.8129									
		CT/MR-PD				0.8450									
Himanshi et al. [33] 2016	PCA and Maximum	CT/MR-T1	Brain			0.8188									
		and CT/MR-PD				0.8390									
	Selection Rule														
J et al. [34]	cascaded PCA and shift														
		MRI/CT	Brain	AANLIB	-	-	-	42.6831	0.0016	0.9253	0.9785	0.0165	5.8625	148.5987	82.9352
Bashir et al. [35] 2019	SWT and PCA	CT and MRI							0.0833		0.1002	−0.1613	5.9796	58.9262	0.2073
		X-ray and MRI	Brain	-	-	-	-	-	0.0425	-	0.9490	0.1450	6.3419	61.8440	0.2259
Ghandour et al. [36] 2024	PCANET and	MRI/CT			3.112971	-	8.419538	37.32781		-	0.614455	-	-	-	-
	nuclear norm	MRI/SPECT	Brain	AANLIB	2.17266	-	4.973007	17.66169	-	0.68963		-	-	-	-
		MRI/PET			2.329743	-	7.51449	26.02398	-	0.680409		-	-	-	-
Daneshvar et al. [37] 2010	IHS and RIM	PET/MRI	Brain	AANLIB	0.6541	-	5.3603	-	-	-	-	-	-	-	-
Haddadpour et al. [87] 2017	IHS and 2DHT	MRI and PET	Brain	AANLIB	-	-	5.227	-	-	-	-	-	-	-	-
Chen [88] 2017	IHS and Log	MRI and PET	Brain	AANLIB	-	-	13.6901	-	-	0.4186	-	-	-	-	6.1858
	Gabor transform														
Dilmaghani [89] 2017	IHS and BEMD	MRI and PET	Brain	-	-	-	5.2793	-	-	-	-	-	3.3402	-	88.4954
Sahu et al. [39] 2014	LP and DCT	CT and MRI	Brain	-	-	-	-	-	-	0.8336	-	-	6.2153	-	49.3859
Li et al. [40] 2019	SR and SML	CT/MR			4.8064	-	-	-	-	0.7858	-	-	-	-	-
		MR-T1/MR-T2	Brain	AANLIB	4.8754	-	-	-	-	0.6255	-	-	-	-	-
	in	PET/MR			4.0235	-	-	-	-	0.7067	-	-	-	-	-
	LP domain	SPECT/MR			5.8928	-	-	-	-	0.7558	-	-	-	-	-
Liu et al. [41] 2020	LP and CSR				3.8976	-	-	-	-	0.7632	-	-	-	-	61.7269
		CT and MRI	Brain	imagefusion	4.8249	-	-	-	-	0.6432	-	-	-	-	69.0794
					3.6948	-	-	-	-	0.6392	-	-	-	-	81.5678
					4.7106	-	-	-	-	0.6039	-	-	-	-	75.4822
Li et al. [42] 2020	LRD framework	MRI-SPECT													
		MRI-PET	Brain	AANLIB	1.6386	-	-	-	-	-	-	-	-	-	64.0980
Wang et al. [16] 2020	LP and ASR				2.7088	-	8.9159	2.6533	-	-	-	-	-	-	8.1978
		CT-MRI	Brain	AANLIB	3.8886	-	10.850	2.7049	-	-	-	-	-	-	8.3676
					4.7934	-	13.5861	3.3301	-	-	-	-	-	-	8.1928
Li et al. [43] 2024	Lp, SR,	Infrared-visible	-	TNO	2.4378		-	-	-	0.5768	-	-	-	-	-
		Multi-focus	-	Lytro	7.4854	-	-	-	-	0.7503	-	-	-	-	-
	and guided filtering	Multi-focus	-	MFI-WHU	8.2343	-	-	-	-	0.7348	-	-	-	-	-
Teng et al. [44] 2010	DWT	CT/MRI			-	-	-	-	-	-	-	-	6.7599	-	-
		PET/MRI	Brain	-	-	-	-	-	-	-	-	-	5.6779	-	-
Bhavana et al. [45] 2015	DWT	MRI and PET			-	-	6.8573	-	0.1911	-	-	-	-	55.3184	-
		MRI and PET	Brain	-	-	-	7.9881	-	0.18589	-	-	-	-	55.4383	-
		MRI and PET			-	-	10.5855	-	0.19144	-	-	-	-	55.3104	-
Soundrapandiyan et al. [46] 2017	IFS and DWT			Indian Scan											
		CT/MRI	Brain	Centre	6.2381	0.9984	-	-	-	-	-	-	6.6815	63.2380	85.3147
Bashir et al. [35] 2019	SWT and PCA	CT and MRI							0.0258	-	0.5871	−0.0955	6.1523	64.0206	0.1419
		X-ray and MRI	Brain	-	-	-	-	-	0.0639	-	0.9027	0.1775	6.2769	60.0758	0.2060
Ganasala et al. [48] 2014	sum of variations														
		CT-MRI													
	and	SPECT-MR	Brain	-	2.92	-	-	-	33.55	-	0.55	-	-	-	85.0
	activity level														
	parameters														
Jin et al. [47] 2018	ICM, S-PCNN,				-	-	7.1237	23.3061		-	-	-	-	-	67.0101
		CT, MRI	Brain	AANLIB	-	-	13.0558	34.6341	-	-	-	-	-	-	86.1786
	and NSST	and PET			-	-	11.6935	32.6684	-	-	-	-	-	-	82.9257
					-	-	15.2545	36.3924	-	-	-	-	-	-	88.1365
Yin et al. [49] 2019	PA-PCNN in NSST domain	CT/MRI											5.1193		87.1653
		MR-T1/MR-T2	Brain	AANLIB									5.1961		80.9326
		MRI/PET											4.9461		62.4796
		MRI/SPECT											4.7940		58.0182
Gai et al. [50] 2019	ISR and EP-PCNN in NSST domain	MR-T1/CT			-	-	-	-	-		-	-	5.3657		86.6892
		MR-T1/MR-PD			-	-	-	-	-	-	-	-	5.1601	-	87.0734
		MR-T1/MR-T2			-	-	-	-	-	-	-	-	5.3589	-	87.6199
		MR-T2/MR-Gad	Brain	AANLIB	-	-	-	-	-	-	-	-	5.2254	-	87.0734
		MRI/SPECT			-	-	-	-	-	-	-	-	4.7277	-	51.6683
		MRI/PET			-	-	-	-	-	-	-	-	5.4490	-	75.8308
Vanitha et al. [51] 2021	SF-PAPCNN in NSST domain	CT/MR			0.7101					0.8102			5.0870		87.358
		MR-T1/MR-T2			0.8144					0.8615			4.5843		81.429
		MRI/PET	Brain	AANLIB	0.8329					0.0453			4.7802		82.162
		MRI/SPECT			0.9248					0.047			4.5266		61.855
Mei et al. [52] 2022	PCNN and SDG in NSST domain	CT/MRI	Brain	-	-	-	-	23.3014	-	0.7213	-	-	6.6191	-	64.3961
Vanitha et al. [53] 2022	PA-PCNN and energy attribute in NSST domain	CT/MR,			0.7051	-	-	-	-	0.6412	-	-	-	-	85.628
		MR-T1/MR-T2	Brain	AANLIB	0.7759	-	-	-	-	0.6939	-	-	-	-	75.345
		MRI/PET			0.771	-	-	-	-	0.511	-	-	-	-	68.19
		MRI/SPECT			0.804	-	-	-	-	0.483	-	-	-	-	64.538
Guo et al. [54] 2023	MI and CSR in NSST	CT/MRI			2.2035	-	-	-	0.8507	-	-	-	5.0790	-	-
		MRI/SPECT	Brain	AANLIB	2.8068	-	-		0.6835	-	-	-	4.9790	-	-
Das et al. [55] 2012	modified SF and PCNN in NSCT	CT/MRI			4.8300	-	-	6.9434	-	0.7771	-	-	6.7724	-	65.8646
		MR-T1/MRA			5.0067	-	-	7.8946	-	0.669	-	-	6.0659	-	68.9896
		CT/MR-GAD	Brain	AANLIB	3.1200	-	-	6.8315	-	0.5180	-	-	4.5234	-	82.3317
		MR-T1/MR-T2			3.4700	-	-	6.9678	-	0.5410	-	-	4.0450	-	79.5945
		CT/MR-PD			3.0593	-	-	6.3261	-	0.5338	-	-	4.3645	-	83.7037
Bhatnagar et al. [56] 2015	directive contrast and normalized Shannon entropy in NSCT				1.8503	0.4725	-	-	-	0.6772	-	-	-	-	-
		CT/MR	Brain		1.4899	0.8142	-	-	-	0.5931	-	-	-	-	-
					3.9493	0.6950	-	-	-	0.6992	-	-	-	-	-
		MR-T1/MR-T2			1.5170	0.8989	-	-	-	0.5691	-	-	-	-	-
Tian et al. [57] 216	NSCT and IPCNN				3.3680	-	-	-	-	0.7324	-	-	7.0255	-	-
		MRI/CT			2.5415	-	-	-	-	0.5048	-	-	5.8632	-	-
		MRI/SPECT	Brain	AANLIB	2.1796	-	-	-	-	0.6384	-	-	4.5399	-	-
		PET/MR-T1			4.0827	-	-	-	-	0.7674	-	-	0.7674	-	-
Zhu et al. [2] 2019	phase congruency and local Laplacian energy in NSCT	CT/MRI			2.2188	-	-	-	-	0.8502	-	-	0.8094	-	-
		MRI/PET	Brain	AANLIB	1.7422	-	-	-	-	0.3475	-	-	0.8059	-	-
		SPECT/MRI			2.6134	-	-	-	-	0.5455	-	-	0.8101	-	-
Sa et al. [21] 2023		CT/MR-T2			2.9673	-	9.8877	-	-	0.5127	-	-	5.0649	63.2085	81.7336
		CT/MR-PD			3.2543	-	9.4748	-	-	0.4704	-	-	5.3986	63.9196	85.0497
		CT/MR-Gad			3.1953	-	9.6266	-	-	0.4736	-	-	4.9821	64.9061	84.4695
	PCNN in NSCT domain	MR-T1/MR-T2	Brain	AANLIB	3.5121	-	12.5236	-	-	0.5861	-	-	4.9101	66.1580	88.2779
		MRI/PET			3.8364	-	11.7520	-	-	0.7837	-	-	-	-	94.3089
		MRI/SPECT			4.0813	-	10.5105	-	-	0.7702	-	-	-	-	80.3603
Javed et al. [69] 2014	Fuzzy transform				1.7912	0.6788	-	-	-	-	-	-	5.738	-	-
		PET/MRI	Brain	AANLIB	1.4292	0.8133	-	-	-	-	-	-	3.5762	-	-
					1.8683	0.6739	-	-	-	-	-	-	5.8204	-	-
Manchanda et al. [68] 2018	Fuzzy transform	MRI/CT		-	-	0.5987	-	-	-	-	-	-	-	-	62.5335
		MRI/CT			-	0.7993	-	-	-	-	-	-	-	-	6.7708
		MRI-T1/MRI-T2			-	0.7178	-	-	-	-	-	-	-	-	75.5276
		MRI-T1/MRI-T2	Brain		-	0.5775	-	-	-	-	-	-	-	-	54.2575
		CT/PET			-	0.8871	-	-	-	-	-	-	-	-	43.9797
		CT/PET			-	0.7878	-	-	-	-	-	-	-	-	86.6075
		MRI/SPECT			-	7838	-	-	-	-	-	-	-	-	53.4464
		MRI/PET			-	0.7435	-	-	-	-	-	-	-	-	81.6643
Yang et al. [70] 2019	Fuzzy transform	MR-GAD/MR-T2			4.9181	-	-	-	-	0.6917	-	-	-	-	-
		CT/MR-T2			3.5558	-	-	-	-	0.6521	-	-	-	-	-
		MR-T1/MR-T2			5.1681	-	-	-	-	0.7344	-	-	-	-	-
		CT/MRI	Brain	AANLIB	5.3025	-	-	-	-	0.8163	-	-	-	-	-
		CT/MR-T2			4.1352	-	-	-	-	0.6315	-	-	-	-	-
		MR-T1/MRA			5.4567	-	-	-	-	0.6895	-	-	-	-	-
		CT/MR-T2			4.2249	-	-	-	-	0.6411	-	-	-	-	-
		MR-GAD/MR-T2			4.1544	-	-	-	-	0.7168	-	-	-	-	-
Xia et al. [60] 2018	SR and PCNN in NSCT domain	CT/MRI			2.2426	0.9567	-	-	-	0.6899	-	-	0.1634	-	55.4061
		MR-PD/MR-T1			2.5474	1.4279	-	-	-	0.5887	-	-	0.9963	-	51.4444
		MR-PD/MR-T2			2.5378	1.7148	-	-	-	0.6530	-	-	0.9768	-	56.6857
		MR-T1/MR-T2	Brain	AANLIB	2.2047	1.2812	-	-	-	0.5732	-	-	0.9956	-	58.7637
		MR-PD/PET			2.7879	-	4.9744	5.9952	-	0.5718	-	-	0.9801	-	67.2682
		MR-T1/PET			2.7559	-	7.5961	6.9475	-	0.5681	-	-	0.9923	-	67.0245
		MR-T2/PET			2.6076	-	7.4678	6.9365	-	0.5451	-	-	0.9769	-	65.1174
Huang et al. [61] 2019	SFLA and PCNN in the IHS and NSCT domains				-	-	-	33.6546	-	-	-	-	3.0621	-	57.2258
		SPECT/CT			-	-	-	20.0956	-	-	-	-	3.9424	-	53.1691
					-	-	-	18.7335	-	-	-	-	3.7399	-	57.1268
					-	-	-	16.4776	-	-	-	-	4.1788	-	58.3122
		SPECT/CT	Brain	AANLIB	-	-	-	17.9095	-	-	-	-	4.837	-	57.775
					-	-	-	27.7654	-	-	-	-	2.658	-	56.3546
					-	-	-	27.1771	-	-	-	-	4.8966	-	66.5782
		SPECT/CT			-	-	-	19.512	-	-	-	-	4.2156	-	60.6457
					-	-	-	22.0022	-	-	-	-	4.2897	-	57.7578
Sa et al. [63] 2023	PAPCNN in NSCT domain	CT/MR			2.8512	-	10.1030	-	-	0.5819	-	-	5.3803	-	-
		CT/MR			3.0729	-	7.2829	-	-	0.6656	-	-	5.0058	-	-
		MR-T1/MR-T2			3.1123	-	10.8864	-	-	0.5871	-	-	5.7306	-	-
		MR-T1/MR-T2	Brain	AANLIB	3.1816	-	10.3080	-	-	0.6230	-	-	5.6301	-	-
		MRI/PET			3.0100	-	8.5367	-	-	0.6805	-	-	4.7006	-	-
		MRI/PET			3.3547	-	11.1658	-	-	0.7425	-	-	5.3461	-	-
		MRI/SPECT			3.4902	-	6.7863	-	-	0.6799	-	-	5.4000	-	-
		MRI/SPECT			2.9615	-	6.8302	-	-	0.7197	-	-	4.5214	-	-
lv et al. [64] 2026	AGPCNN, fractal dimension, and NSCT	Multi-focus	-	Lytro	6.8351	-	-	-	-	0.7307	-	-	-	35.0579	-
			-	MFI-WHU	7.0174	-	-	-	-	0.7136	-	-	-	35.3750	-
li et al. [65] 2025	Adaptive dual PCNN and Curvelet	Multi-focus	-	Lytro	6.7296	-	-	-	22.2336	0.7285	-	-	-	35.0684	-
				MFFW	5.1029	-	-	-	43.0355	0.6290	-	-	-	32.5370	-
				MFI-WHU	7.5215	-	-	-	20.5981	0.7199	-	-	-	35.3640	-
Lv et al. [58] 2025	A dual-channel Rybak NN and NSCT		-	Lytro	7.0950	-	-	-	21.4427	0.7479	-	-	-	35.2352	-
		Multi-focus		MFFW	5.8296	-	-	-	40.0503	0.7153	-	-	-	33.0138	-
					MFI-WHU	7.9063	-	-	20.3050	0.7300	-	-	-	35.4395	-
Lv et al. [66] 2023	PAPCNN, fractal dimension, and NSST		-	Lytro	6.8598	-	11.7205	-	27.0097	0.7384	-	-	-	34.2875	-
		Multi-focus													
Li & Wu [72] 2019	Dense Auto-encoder	IR+Visible													
				TNO / RoadScene	-	0.7211	-	-	-	0.4761	-	-	6.8419	59.953$\pm$1.99	-
Liu et al. [17] 2017	CNN in LP domain	CT/MR													
		MR-Gad/MR-T1	Brain	AANLIB	-	-	-	-	-	0.6309	-	-	6.1741	-	-
		MR-T1/MR-T2													
		MR-T2/SPECT													
Hermessi et al. [20] 2018	Local energy and CNN in NSST domain				5.7545	-	-	7.6877	-	-	-	-	6.7612	-	64.2170
		CT/MRI	Brain	-	3.4492	-	-	8.8984	-	-	-	-	5.5572	-	80.4658
Xia et al. [75] 2019	largest local variance and local energy in DCNN domain	CT/MRI	Abdomen		5.147	-	7.112	13.640	-	-	-	-	7.176	-	45.907
		CT/PET	Abdomen	AANLIB	6.344	-	6.545	11.354	-	-	-	-	7.622	-	75.422
		CT/MRI	Brain		3.464	-	3.395	8.031	-	-	-	-	6.188	-	21.386
Ding et al. [4] 2020	The activity level measurement, the weight map, the initial weight, and the CNN in NSST domain				3.2722	-	7.1552	-	-	-	-	-	-	-	57.9205
					4.2751	-	10.3155	-	-	-	-	-	-	-	69.1393
		CT/MRI	Brain	AANLIB	3.3678	-	7.7668	-	-	-	-	-	-	-	77.2744
					3.3421	-	6.1253	-	-	-	-	-	-	-	101.008
					3.5295	-	16.7255	-	-	-	-	-	-	-	86.9107
					3.7677	-	8.8360	-	-	-	-	-	-	-	73.7021
Wang et al. [76] 2021	PHF-CNN in NSCT domain	CT/MR-GAD			2.1460	0.7269	-	-	-	0.6449	-	-	-	-	-
		CT/MR-T1			2.1713	0.8704	-	-	-	0.6215	-	-	-	-	-
		CT/MR-T2	Brain		2.4991	0.6232	-	-	-	0.7910	-	-	-	-	-
		MR-T1/MR-T2		-	2.4774	0.6642	-	-	-	0.6289	-	-	-	-	-
		MR-T1/MR-GAD			2.4489	0.6919	-	-	-	0.6495	-	-	-	-	-
Li et al. [77] 2021	Supervisied Learning in CNN domain	CT/MRI	Brain	-	-	-	-	-	-	0.8911	-	-	0.8002	-	-
		MRI/SPECT			-	-	-	-	-	0.2164	-	-	0.1177	-	-
El-Shafai et al. [78] 2023	CNN	CT/MR	Brain	-	0.917	0.526	-	-	-	0.497	-	-	7.620	-	0.301
					0.904	0.462	-	-	-	0.760	-	-	6.542	-	0.296
Li et al. [79] 2024	Coupled neural P systems and fractal dimension + NSCT		-	Lytro	6.9683	-	-	-	-	0.7390	-	-	-	-	-
		Multi-focus		MFI-WHU	7.8107	-	-	-	-	0.7296	-	-	-	-	-
				MFFW	5.0505	-	-	-	-	0.6377	-	-	-	-	-
Zhang et al. [80] 2020	CNN	multi-focus						2.886	19.42						
			Medical	Harvard	-		-	3.402	24.98		-	-			-
			IR + visible	TNO				1.865	11.31						
Xu et al. [81] 2021	A Unified Unsupervised CNN	multi-focus													
			Medical	Harvard	-		1.9921$\pm$0.002				-	-		63.458$\pm$1.15	-
			IR + visible	TNO			1.9909$\pm$0.005							62.914$\pm$2.07	
Ma et al. [82] 2019	GAN			TNO			1.9824$\pm$0.008							60.535$\pm$1.98	
			IR + visible	RoadScene	-		1.9850$\pm$0.009				-	-		61.341$\pm$2.59	-
Ma et al. [83] 2020	Dual-Discriminator GAN			TNO			1.9824$\pm$0.006							60.248$\pm$1.49	
			IR + visible	RoadScene	-		1.9805$\pm$0.009				-	-		60.051$\pm$2.32	-

This table presents fusion results across seven major method categories: PCA/IHS-based methods, DWT/SWT-based methods, NSCT/PCNN-based methods, CNN-based methods, GAN-based methods, autoencoder-based methods, and fuzzy transform methods. For each category, multiple quantitative metrics (MI, SSIM, AG, SF, MSE, QAB/F, NCC, AD, EN, PSNR, SD) are reported, enabling direct comparison of how different techniques impact the resulting fused images. This table contains extensive numerical outcomes. Key values: MI ranges 0.65–7.52 (best: 7.52 by Li et al., 2025); SSIM ranges 0.47–0.9984 (best: 0.9984 by Soundrapandiyan et al., 2017); QAB/F ranges 0.04–0.93 (best: 0.9253 by J et al., 2018); PSNR ranges 55–149 dB (best: 148.60 dB by J et al., 2018); EN ranges 3.06–7.76 (best: 7.62 by El-Shafai et al., 2023; DenseFuse: 6.84).

## Challegences in MIF area

The diverse nature of medical imaging modalities, such as CT, MRI, PET, and SPECT, faces many fundamental challenges for MIF. A common issue is achieving accurate registration between source images from different modalities with varying resolutions. Furthermore, it is crucial to retain the complementary information provided by these modalities. For example, one must preserve the CT images’ high spatial resolution while combining the MRI soft tissue characteristics.

In medical imaging, the noise and artifacts may reduce the fusion accuracy and hide crucial details. Efficient algorithms that can merge multimodal images without causing distortions or eliminating clinical information are essential. Furthermore, the efficiency of the computation is also a challenge, in real-time applications such as surgery or diagnosis. Finally, there are no uniform evaluation metrics for assessing fusion quality since different medical applications highlight various factors such as contrast, structural clarity, or accuracy. Overcoming such challenges is crucial to enhancing diagnostic accuracy and improving patient outcomes in healthcare.

## Machine learning (ML) applications in MIF area

MIF involves combining two or more images from different modalities to improve the accuracy of the final output. MIF applications commonly utilize ML to enhance the quality of the fused images. MIF has numerous ML applications, such as the enhancement of medical image visualization. ML can utilize feature-based fusion that extract features from medical images, leading to an understanding of the fused images. ML is mainly used in image registration, which aligns multiple medical images of the same scene captured by several sensors. This process significantly enhances medical image analysis.Another application involves detecting tumors from medical images by enhancing image characteristics to identify tumors for several organs, improve the accuracy of tumor detection, and aid in the optimal treatment of diseases. The fused images can help doctors specially surgeons in the surgical operations to improve the patient outcomes and make thses more precision.

## Conclusion

Medical image fusion (MIF) combines two multimodality medical images to improve the performance of the final output image. The ideal fusion method has a short computational time with high spatial and color resolution. The resulting image is effective for accurate patient diagnosis and treatment. We illustrate MIF methods in detail, including DWT, PCA, NSCT, NSST, CNN, and PCNN. These methods are compared to determine the best fusion method, particularly for medical images. We also cover MMIF examples and various combinations of medical images, which are summarized. Finally, the main performance metrics are evaluated to show the fused image quality.

## Data Availability

The data used in this study are acquired from K. A. Johnson and J. A. Becker, The Whole Brain Atlas, 2024. URL: http://www.med.harvard.edu/aanlib/, (accessed 19 November 2024).

## Abbreviations

2DHT: Two-Dimensional Hilbert Transform
AG: Average Gradient
AGPCNN: Adaptive parameter-controlled PCNN
ASR: Adaptive Sparse Representation
BEMD: Bidimensional Empirical Mode Decomposition
CB: Chen & Blum metric
CEUS: Contrast-Enhanced Ultrasound
CNN: Convolutional Neural Networks
CS: Compressed Sensing
CSR: Convolutional Sparse Representation
CT: Computed Tomography
DCT: Discrete Cosine Transform
DCNN: Deep Convolutional Neural Networks
DTCWT: Dual-Tree Complex Wavelet Transformations
DWT: Discrete Wavelet Transform
DWI: Diffusion-weighted imaging
EN: Entropy
EP-PCNN: Edge Preservation-Pulse Coupled Neural Network
FLAIR: Fluid-Attenuated Inversion Recovery
FF: Fusion Factor
GAN: Generative Adversarial Network
GB: GigaByte
GIF: Guided Image Filtering
HSV: Hue-Saturation-Value
ICM: Intersecting Cortical Models
IFS: Intuitionistic Fuzzy Sets
IHS: Intensity Hue Saturation
IPCNN: Improved Pulse-Coupled Neural Network
IQI: Image Quality Index
ISR: Improved Sparse Representation
LP: Laplacian Pyramid
LRD: Laplacian Re-Decomposition
MI: Mutual Information
MIF: Medical Image Fusion
MRI: Magnetic resonance imaging
MSE: Mean Square Error
MST: MultiScale Transform
NOD: Non-Overlapping Domain
NSCT: Non-Subsampled Contourlet Transform
NSCT-PCLP: NonSubsampled Contourlet Transform - phase congruency local Laplacian
NSLP: Non-Subsampled Laplacian Pyramid
NSPFB: Non-Subsampled Pyramid Filter Bank
NSDFB: Non-Subsampled Directional Filter Bank
NSST: Non-Subsampled Shearlet Transform
OD: Overlapping Domain
OCT: Optical Coherence Tomography
PAPCNN: Parameter-Adaptive Pulse-Coupled Neural Network
PCA: Principal Component Analysis
PCANET: Principal Component Analysis network
PCNN: Pulse Coupled Neural Network
PET: Positron Emission Tomography
PHF-CNN: Perceptual High-Frequency CNN
PSNR: Peak Signal to Noise Ratio
RIM: Retina-Inspired Model
RMSE: Root Mean Square Error
SD: Standard Deviation
SDG: Sum of Directional Gradients
SF: Spatial Frequency
SFLA: Shuffled Frog Leaping Algorithm
ShF: Shearlet transform Filter
SML: Sum-Modified Laplacian
S-PCNN: Simplified Pulse-Coupled Neural Network
SPECT: Single-Photon Emission Computed Tomography
SR: Sparse Representation
SPD: Structural Patch Decomposition
SSIM: Structural Similarity Index
SWT: Stationary Wavelet Transform
TB: TeraByte
Ultrasound: US
WSEML: Weighted Sum of Eight neighborhood-based Modified Laplacian
